# Single-pot glycoprotein biosynthesis using a cell-free transcription-translation system enriched with glycosylation machinery

**DOI:** 10.1038/s41467-018-05110-x

**Published:** 2018-07-12

**Authors:** Thapakorn Jaroentomeechai, Jessica C. Stark, Aravind Natarajan, Cameron J. Glasscock, Laura E. Yates, Karen J. Hsu, Milan Mrksich, Michael C. Jewett, Matthew P. DeLisa

**Affiliations:** 1 Robert Frederick Smith School of Chemical and Biomolecular Engineering, Cornell University, Ithaca, NY 14853 USA; 2 Department of Chemical and Biological Engineering, Northwestern University, Evanston, IL 60208 USA; 3 Chemistry of Life Processes Institute, 2170 Campus Drive, Evanston, IL 60208-3120 USA; 4 Center for Synthetic Biology, Northwestern University, 2145 Sheridan Road, Evanston, IL 60208-3120 USA; 5 Department of Microbiology, Cornell University, Ithaca, NY 14853 USA; 6 Department of Mechanical Engineering, Northwestern University, 2145 Sheridan Rd Technological Institute B224, Evanston, IL 60208-3120 USA; 7 Department of Chemistry, Northwestern University, Evanston, IL 60208 USA; 8 Department of Cell and Molecular Biology, Northwestern University, Chicago, IL 60611 USA; 9 Department of Biomedical Engineering, Northwestern University, Evanston, IL 60208 USA

## Abstract

The emerging discipline of bacterial glycoengineering has made it possible to produce designer glycans and glycoconjugates for use as vaccines and therapeutics. Unfortunately, cell-based production of homogeneous glycoproteins remains a significant challenge due to cell viability constraints and the inability to control glycosylation components at precise ratios in vivo. To address these challenges, we describe a novel cell-free glycoprotein synthesis (CFGpS) technology that seamlessly integrates protein biosynthesis with asparagine-linked protein glycosylation. This technology leverages a glyco-optimized *Escherichia coli* strain to source cell extracts that are selectively enriched with glycosylation components, including oligosaccharyltransferases (OSTs) and lipid-linked oligosaccharides (LLOs). The resulting extracts enable a one-pot reaction scheme for efficient and site-specific glycosylation of target proteins. The CFGpS platform is highly modular, allowing the use of multiple distinct OSTs and structurally diverse LLOs. As such, we anticipate CFGpS will facilitate fundamental understanding in glycoscience and make possible applications in on demand biomanufacturing of glycoproteins.

## Abstract (web summary)

The ability to produce homogeneous glycoproteins is expected to advance fundamental understanding in glycoscience, but current in vivo-based production systems have several limitations. Here, the authors develop an *E. coli* extract-based one-pot system for customized production of *N*-linked glycoproteins.

## Introduction

Asparagine-linked (*N*-linked) protein glycosylation is one of the most common post-translational modifications in eukaryotes, and profoundly affects protein properties such as folding, stability, immunogenicity, and pharmacokinetics^1–3^. The attached *N-*glycans can participate in a wide spectrum of biological processes such as immune recognition/response^4,5^ and stem cell fate^6^. Moreover, the intentional engineering of protein-associated glycans can be used to manipulate protein therapeutic properties such as enhancing in vivo activity and half-life^7^.

At present, however, the inherent structural complexity of glycans and the corresponding difficulties producing homogeneously glycosylated proteins have slowed advances in our understanding of glycoprotein functions and limited opportunities for biotechnological applications. Moreover, because glycan biosynthesis is neither template-driven nor genetically encoded, glycans cannot be produced from recombinant DNA technology. Instead, *N-*glycans are naturally made by coordinated expression of multiple glycosyltransferases (GTs) across several subcellular compartments. This mode of biosynthesis combined with the lack of a strict proofreading system results in inherent glycan heterogeneity and accounts for the large diversity of structures in the expressed glycan repertoire of a cell or organism^8,9^. Further complicating matters is the paucity of structure–function relationships for GTs, which hinders a priori prediction of glycan structure. Altogether, these factors have frustrated production of homogeneous glycans and glycoconjugates in biological systems and restricted our capacity to elucidate the biochemical and biophysical effects of glycans on the proteins to which they are attached. Thus, there is an unmet need for a technology capable of rapidly producing useful quantities of proteins featuring user-specified glycosylation for biochemical and structural biology studies.

Recent pioneering efforts in glycoengineering of cellular systems including mammalian^10^, yeast^11^, and bacterial cells^12^ have expanded our ability to reliably synthesize chemically defined glycans and glycoproteins. Despite the promise of these systems, protein expression yields often remain low and design-build-test (DBT) cycles—iterations of re-engineering organisms to test new sets of enzymes—can be slow. One promising alternative to cell-based systems is cell-free protein synthesis (CFPS) in which protein synthesis occurs in vitro without using intact, living cells. Recently, a technical renaissance has revitalized CFPS systems to help meet increasing demands for simple and efficient protein synthesis, with *Escherichia coli*-based CFPS systems now exceeding grams of protein per liter reaction volume^13^, with the ability to support co- or post-translational modifications^14–18^. As a complement to in vivo expression systems, cell-free systems offer several potential advantages. First, the open nature of the reaction allows the user to directly influence biochemical systems of interest. As a result, new components can be added or synthesized, and maintained at precise concentrations^19,20^. Second, cell-free systems bypass viability constraints making possible the production of proteins at titers that would otherwise be toxic in living cells^21^. Third, processes that take days or weeks to design, prepare, and execute in vivo can be done more rapidly in a cell-free system^22,23^, leading to high-throughput production campaigns on a whole-proteome scale^24^ with the ability to automate^25^.

Unfortunately, CFPS systems have been limited by their inability to co-activate efficient protein synthesis and glycosylation. The best characterized and most widely adopted CFPS systems use *E. coli* lysates to activate in vitro protein synthesis, but these systems are incapable of making glycoproteins because *E. coli* lacks endogenous glycosylation machinery. Glycosylation is possible in some eukaryotic CFPS systems, including those prepared from insect cells^26^, trypanosomes^27^, hybridomas^28^, or mammalian cells^29–31^. However, these platforms are limited to endogenous machinery for performing glycosylation, meaning that (i) the possible glycan structures are restricted to those naturally synthesized by the host cells and (ii) the glycosylation process is carried out in a black box and thus difficult to engineer or control. Additionally, eukaryotic CFPS systems are technically difficult to prepare, often requiring supplementation with microsomes^31–33^, and suffer from inefficient protein synthesis and glycosylation yields due to inefficient trafficking of nascent polypeptide chains to microsomes^27,33^.

Despite progress in eukaryotic cell-free systems, cell-free extracts from bacteria like *E. coli* offer a blank canvas for studying glycosylation pathways, provided they can be activated in vitro. A recent work from our group highlights the ability of CFPS to enable glycoprotein synthesis in bacterial cell-free systems by augmenting commercial *E. coli*-based cell-free translation systems with purified components from a bacterial *N*-linked glycosylation pathway^34^. While these results established the possibility of *E. coli* lysate-based glycoprotein production, there are several drawbacks of using purified glycosylation components that limit system utility. First, preparation of the glycosylation components required time-consuming and cost-prohibitive steps, namely purification of a multipass transmembrane oligosaccharyltransferase (OST) enzyme and organic solvent-based extraction of lipid-linked oligosaccharide (LLO) donors from bacterial membranes. These steps significantly lengthen the process development timeline, requiring 3–5 days each for preparation of the LLO and OST components, necessitate skilled operators and specialized equipment, and result in products that must be refrigerated and are stable for only a few months to a year. Second, glycoproteins were produced using a sequential translation/glycosylation strategy, which required 20 h for cell-free synthesis of the glycoprotein target and an additional 12 h for post-translational protein glycosylation.

Here, we addressed these drawbacks by developing an integrated cell-free glycoprotein synthesis (CFGpS) technology that bypasses the need for purification of OSTs and organic solvent-based extraction of LLOs. The creation of this streamlined CFGpS system was made possible by two important discoveries: (i) crude extract prepared from the glyco-optimized *E. coli* strain, CLM24, is able to support cell-free protein expression and *N-*linked glycosylation; and (ii) OST- and LLO-enriched extracts derived from CLM24 are able to reproducibly co-activate protein synthesis and *N-*glycosylation in a reaction mixture that minimally requires priming with DNA encoding the target glycoprotein of interest. Importantly, the CFGpS system decouples production of glycoprotein synthesis components (i.e., OSTs, LLOs, translation machinery) and the glycoprotein target of interest, providing significantly reduced cell viability constraints compared to in vivo systems. The net result is a one-pot bacterial glycoprotein biosynthesis platform whereby different acceptor proteins, OSTs, and/or oligosaccharide structures can be functionally interchanged and prototyped for customizable glycosylation.

## Results

### Efficient CFGpS using extracts from glyco-optimized chassis strain

**Fig. 1:**
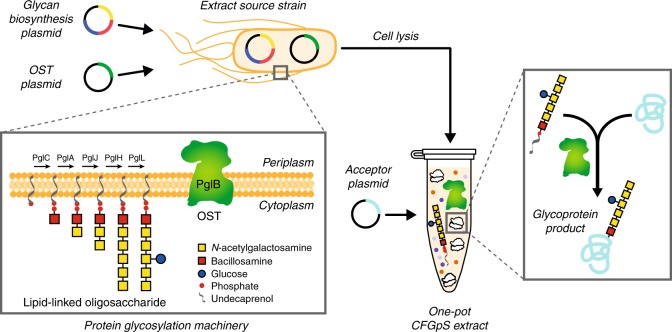
Schematic of single-pot CFGpS technology. Glycoengineered *E. coli* that are modified with (i) genomic mutations that benefit glycosylation reactions and (ii) plasmid DNA for producing essential glycosylation components (i.e., OSTs, LLOs) serve as the source strain for producing crude S30 extracts. Candidate glycosylation components can be derived from all kingdoms of life and include single-subunit OSTs like *C. jejuni* PglB and LLOs bearing *N*-glycans from *C. jejuni* that are assembled on Und-PP by the Pgl pathway enzymes. Following extract preparation by lysis of the source strain, one-pot biosynthesis of *N-*glycoproteins is initiated by priming the extract with DNA encoding the acceptor protein of interest

To develop a one-pot glycoprotein synthesis system, the bacterial protein glycosylation locus (*pgl*) present in the genome of the Gram-negative bacterium *Campylobacter jejuni* was chosen as a model glycosylation system (Fig. 1). This gene cluster encodes an asparagine-linked (*N-*linked) glycosylation pathway that is functionally similar to that of eukaryotes and archaea^35^, involving a single-subunit OST, PglB, that catalyzes the en bloc transfer of a preassembled 1.4 kDa GlcGalNAc_5_Bac heptasaccharide (where Bac is bacillosamine) from the lipid carrier undecaprenyl pyrophosphate (Und-PP) onto asparagine residues in a conserved motif (D/E-X_−__1_-N-X_+1_-S/T, where X_−1_ and X_+1_ are any residues except proline) within acceptor proteins. PglB was selected because we previously showed that *N-*glycosylated acceptor proteins were reliably produced when cell-free translation kits were supplemented with (i) *C. jejuni* PglB (*Cj*PglB) purified from *E. coli* cells and (ii) LLOs extracted from glycoengineered *E. coli* cells expressing the enzymes for producing the *C. jejuni N*-glycan on Und-PP (*Cj*LLOs)^34^. Additionally, PglB has been used in engineered *E. coli* for transferring eukaryotic trimannosyl chitobiose glycans (mannose_3_-*N*-acetylglucosamine_2_, Man_3_GlcNAc_2_) to specific asparagine residues in target proteins^12^.

Establishing a CFGpS system first required crude cell extracts suitable for glycoprotein synthesis; hence, we selected *E. coli* strain CLM24 that was previously optimized for in vivo protein glycosylation^36^. CLM24 has two attributes that we hypothesized would positively affect cell-free protein glycosylation. First, CLM24 does not synthesize *O*-polysaccharide antigen due to an inactivating insertion in w*bbL*, which encodes a rhamnosyl transferase that transfers the second sugar of the O16 subunit to Und-PP^37^. Thus, absence of WbbL should allow uninterrupted assembly of engineered glycans, such as the *C. jejuni* heptasaccharide, on Und-PP. Second, CLM24 cells lack the *waaL* gene, which encodes the ligase that transfers *O*-polysaccharide antigens from Und-PP to lipid A-core. Because WaaL can also promiscuously transfer engineered glycans that are assembled on Und-PP^12,38^, the absence of this enzyme should favor accumulation of target glycans on Und-PP.

**Fig. 2:**
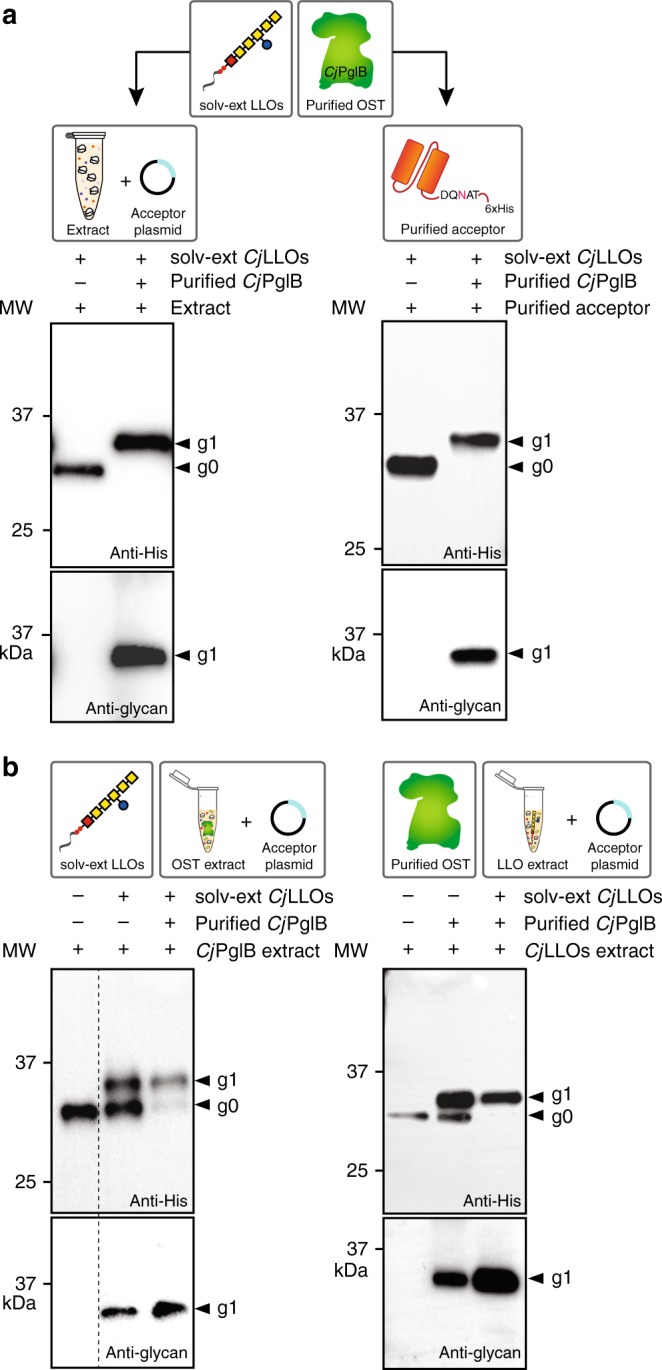
Extract from glyco-optimized chassis strain supports CFGpS. **a** (left) Western blot analysis of scFv13-R4^DQNAT^ produced by crude CLM24 extract supplemented with purified *Cj*PglB and organic solvent-extracted (solv-ext) *Cj*LLOs, and primed with plasmid pJL1-scFv13-R4^DQNAT^. (right) Western blot analysis of in vitro glycosylation reaction using purified scFv13-R4^DQNAT^ acceptor protein that was incubated with purified *Cj*PglB and organic solvent-extracted (solv-ext) *Cj*LLOs. Control reactions (lane 1 in each panel) were performed by omitting purified *Cj*PglB. **b** (left) Western blot analysis of scFv13-R4^DQNAT^ produced by crude CLM24 extract selectively enriched with *Cj*PglB from heterologous overexpression from pSF-*Cj*PglB. (right) Western blot analysis of scFv13-R4^DQNAT^ produced by crude CLM24 extract selectively enriched with *Cj*LLOs from heterologous overexpression from pMW07-pglΔB. Reactions were primed with plasmid pJL1-scFv13-R4^DQNAT^ and supplemented with purified *Cj*PglB and organic solvent-extracted (solv-ext) *Cj*LLOs as indicated. Control reactions (lane 1 in each panel) were performed by omitting solv-ext *Cj*LLOs in (left) or purified *Cj*PglB (right) in (**b**). Blots were probed with anti-hexa-histidine antibody (anti-His) to detect the acceptor protein or hR6 serum (anti-glycan) to detect the *N-*glycan. Arrows denote aglycosylated (g0) and singly glycosylated (g1) forms of scFv13-R4^DQNAT^. Molecular weight (MW) markers are indicated at left. Results are representative of at least three biological replicates

To determine whether CLM24 could be used as a chassis strain to support integrated cell-free transcription, translation, and glycosylation, we first prepared crude S30 extract from these cells using a rapid and robust procedure for extract preparation based on sonication^39^. Then, 15-μL batch-mode, sequential CFGpS reactions were performed using CLM24 crude extract that was supplemented with the following: (i) an OST catalyst in the form of purified *Cj*PglB^34^; (ii) oligosaccharide donor in the form of *Cj*LLOs that were isolated by organic solvent extraction from the membrane fraction of glycoengineered *E. coli* cells ^34^; and (iii) plasmid DNA encoding the model acceptor protein scFv13-R4^DQNAT^, an anti-β-galactosidase (β-gal) single-chain variable fragment (scFv) antibody modified C-terminally with a single DQNAT motif^12^. The glycosylation status of scFv13-R4^DQNAT^ was analyzed by SDS-PAGE and immunoblotting with an anti-polyhistidine (anti-His) antibody or hR6 serum that is specific for the *C*. *jejuni* heptasaccharide glycan^40^. Following an overnight reaction at 30 °C, highly efficient glycosylation was achieved as evidenced by the mobility shift of scFv13-R4^DQNAT^ entirely to the mono-glycosylated (g1) form in anti-His immunoblots and the detection of the *C. jejuni* glycan attached to scFv13-R4^DQNAT^ by hR6 serum (Fig. 2a). For synthesis of scFv13-R4^DQNAT^, the reaction mixture was modified to be oxidizing, through the addition of iodoacetamide and a 3:1 ratio of oxidized and reduced glutathione, demonstrating the flexibility of CFGpS reaction conditions for producing eukaryotic glycoprotein targets. The efficiency achieved in this CFGpS system rivaled that of an in vitro glycosylation reaction in which the scFv13-R4^DQNAT^ acceptor protein was expressed and purified from *E. coli*, and then incubated overnight with purified *Cj*PglB and extracted *Cj*LLOs (Fig. 2a). As expected, when *Cj*PglB was omitted from the reaction, the scFv13-R4^DQNAT^ acceptor protein was produced only in the aglycosylated (g0) form. The results generated here with CLM24 extract are consistent with our earlier studies using an *E. coli* S30 extract-based CFPS system or purified translation machinery^34^, and establish that the *C. jejuni N*-linked protein glycosylation mechanism can be functionally reconstituted outside the cell.

### Expanding the glycan repertoire of cell-free glycosylation

**Fig. 3:**
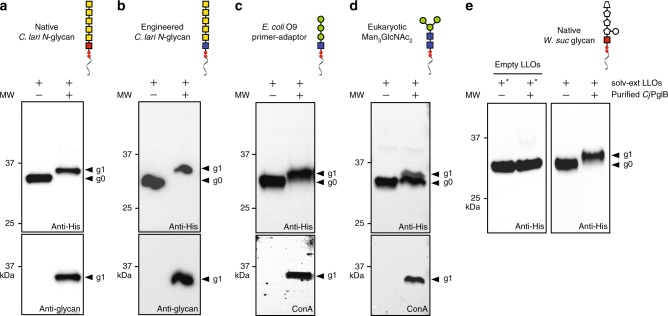
Expanding cell-free glycosylation with different oligosaccharide structures. Western blot analysis of in vitro glycosylation reaction products generated with purified scFv13-R4^DQNAT^ acceptor protein, purified *Cj*PglB, and organic solvent-extracted (solv-ext) LLOs from cells carrying: **a** plasmid pACYC*pgl4* for making the native *C. lari* hexasaccharide *N-*glycan; **b** plasmid pACYC*pgl2* for making the engineered *C. lari* hexasaccharide *N-*glycan; **c** plasmid pO9-PA for making the *E. coli* O9 ‘primer-adaptor’ Man_3_GlcNAc structure; **d** plasmid pConYCGmCB for making the eukaryotic Man_3_GlcNAc_2_ *N-*glycan structure; and **e** fosmid pEpiFOS-5*pgl5* for making the native *W. succinogenes* hexasaccharide *N-*glycan. Reactions were run at 30 °C for 16 h. Blots were probed with anti-His antibody to detect the acceptor protein and one of the following: hR6 serum that cross-reacts with the native and engineered *C. lari* glycans or ConA lectin that binds internal and non-reducing terminal α-mannosyl groups in the Man_3_GlcNAc and Man_3_GlcNAc_2_ glycans. Because structural determination of the *W. succinogenes N-*glycan is currently incomplete, and because there are no available antibodies, the protein product bearing this *N-*glycan was only probed with the anti-His antibody. As an additional control for this glycan, we included empty LLOs prepared from the same host strain but lacking the pEpiFOS-5*pgl5* fosmid (left hand panel, “+” signs marked with an asterisk). Arrows denote aglycosylated (g0) and singly glycosylated (g1) forms of the scFv13-R4^DQNAT^ protein. Molecular weight (MW) markers are indicated at left. Results are representative of at least three biological replicates

To date, only the *C. jejuni* glycosylation pathway has been reconstituted in vitro^34^, and it remains an open question whether our system can be reconfigured with different LLOs and OSTs. Therefore, to extend the range of glycan structures beyond the *C. jejuni* heptasaccharide, we performed glycosylation reactions in which the solvent-extracted *Cj*LLOs used above were replaced with oligosaccharide donors extracted from *E. coli* cells carrying alternative glycan biosynthesis pathways. These included LLOs bearing the following glycan structures: (i) native *C. lari* hexasaccharide *N*-glycan^40^; (ii) engineered GalNAc_5_GlcNAc based on the *Campylobacter lari* hexasaccharide *N-*glycan^41^; (iii) native *Wolinella succinogenes* hexasaccharide *N*-glycan containing three 216-Da monosaccharides and an unusual 232-Da residue at the non-reducing end^42^; (iv) engineered *E. coli* O9 primer-adaptor glycan, Man_3_GlcNAc, that links the O-chain and core oligosaccharide in the lipopolysaccharide of several *E. coli* and *Klebsiella pneumoniae* serotypes^43^; and (v) eukaryotic trimannosyl core *N-*glycan, Man_3_GlcNAc_2_^12^. Glycosylation of scFv13-R4^DQNAT^ with each of these different glycans was observed to occur only in the presence of *Cj*PglB (Fig. 3). It should be noted that 100% glycosylation conversion was observed for each of these glycans except for the Man_3_GlcNAc_2_ *N-*glycan, which had a conversion of ~40% as determined by densitometry analysis. While the reasons for this lower efficiency remain unclear, conjugation efficiency of the same Man_3_GlcNAc_2_ glycan to acceptor proteins in vivo was reported to be even lower (<5%)^12,44^. Hence, transfer of Man_3_GlcNAc_2_ to acceptor proteins in vitro appears to overcome some of the yet-to-be-identified bottlenecks of in vivo glycosylation. This result is likely due to the opportunity with CFGpS to control the concentration of reaction components, for example, providing a higher local concentration of LLO donors. Importantly, scFv13-R4^DQNAT^ was uniformly decorated with a Man_3_GlcNAc_2_ glycan as evidenced by liquid chromatography-mass spectrometry (LC–MS). Specifically, the only major glycopeptide product to be detected was a triply-charged ion containing an *N*-linked pentasaccharide with *m*/*z* = 1032.4583, consistent with the Man_3_GlcNAc_2_ glycoform (Supplementary Fig. 1). The tandem MS spectra for this triply-charged glycopeptide yielded an excellent y-ion series and a good b-ion series enabling conclusive determination of the tryptic glycopeptide sequence and attachment of the Man_3_GlcNAc_2_ glycoform at residue N273 of the scFv13-R4^DQNAT^ protein (Supplementary Fig. 2). Taken together, these results demonstrate that structurally diverse glycans, including those that resemble eukaryotic structures, can be modularly interchanged in cell-free glycosylation reactions.

### Extracts enriched with OSTs or LLOs co-activate glycosylation

To circumvent the need for exogenous addition of purified glycosylation components, we hypothesized that heterologous overexpression of OST or GT enzymes directly in the chassis strain would yield extracts that are selectively enriched with the requisite glycosylation components. This strategy was motivated by a recent metabolic engineering approach whereby multiple cell-free lysates were each selectively enriched with an overexpressed metabolic enzyme and then combinatorially mixed to construct an intact pathway^20,22^. However, a fundamental difference in our system is the fact that the OST and LLOs are not soluble components but instead reside natively in the inner cytoplasmic membrane. This is potentially problematic because of the significant breakup of the cell membrane during S30 extract preparation. However, it has been established that fragments of the *E. coli* inner membrane reform into membrane vesicles, some of which are inverted but others that are orientated properly^45^, and thus could supply the OST and LLOs in a functionally accessible conformation within the extract.

To test this hypothesis, we used a high-pressure homogenization method to prepare crude S30 extract from CLM24 cells carrying a plasmid-encoded copy of *Cj*PglB such that the resulting cell-free lysates were selectively enriched with detectable quantities of full-length OST enzyme as confirmed by Western blot analysis (Supplementary Fig. 3a). Similarly, crude S30 extract from CLM24 cells overexpressing the *C. jejuni* glycan biosynthesis enzymes produced lysate that was selectively enriched with *Cj*LLOs as confirmed by dot blot analysis with hR6 serum (Supplementary Fig. 3b). It should be noted that the amount of *Cj*LLOs enriched in the crude extract rivaled that produced by the significantly more tedious organic solvent extraction method. Importantly, when 15-μL batch-mode sequential CFGpS reactions were performed using the OST-enriched crude extract that was supplemented with solvent-extracted *Cj*LLOs and plasmid DNA encoding scFv13-R4^DQNAT^, clearly detectable glycosylation of the acceptor protein was observed (Fig. 2b). The conversion of acceptor protein to glycosylated product was ~50%; however, further supplementation with purified *Cj*PglB increased the conversion to nearly 100%, indicating that the amount of OST in the crude extract might have been limiting under the conditions tested. When similar CFGpS reactions were performed using the *Cj*LLO-enriched crude extract supplemented with purified *Cj*PglB and plasmid DNA encoding scFv13-R4^DQNAT^, >80% glycosylation of the acceptor protein was observed, which reached 100% when additional donor glycans were supplemented (Fig. 2b).

### Modularity enables glycosylation components to be interchanged

**Fig. 4:**
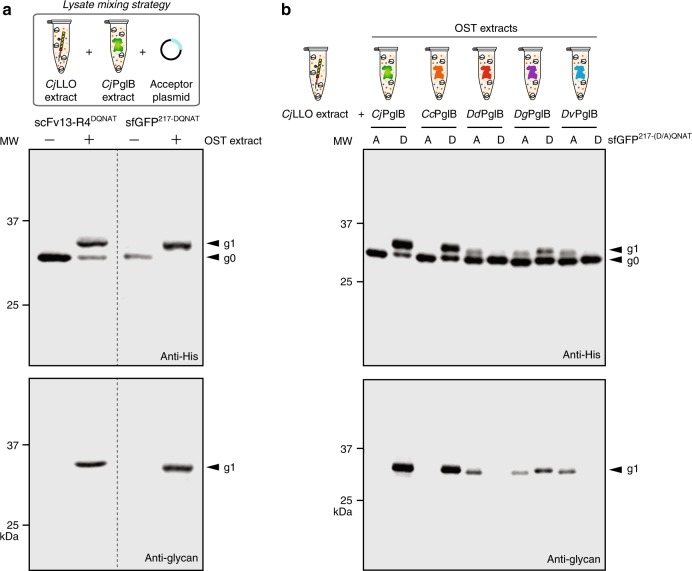
Mixing of CFGpS extracts enables rapid prototyping of different OST enzymes. **a** Western blot analysis of CFGpS reactions performed using lysate mixing strategy whereby *Cj*LLO lysate derived from CLM24 cells carrying pMW07-pglΔB was mixed with *Cj*PglB lysate derived from CLM24 cells carrying pSF-*Cj*PglB, and the resulting CFGpS mixture was primed with plasmid DNA encoding either scFv13-R4^DQNAT^ or sfGFP^217-DQNAT^. **b** Western blot analysis of CFGpS reactions performed using *Cj*LLO lysate mixed with extract derived from CLM24 cells carrying a pSF plasmid encoding one of the following OSTs: *Cj*PglB, *Cc*PglB, *Dd*PglB, *Dg*PglB, or *Dv*PglB. Mixed lysates were primed with plasmid DNA encoding either sfGFP^217-DQNAT^ (D) or sfGFP^217-AQNAT^ (A). Blots were probed with anti-His antibody to detect the acceptor proteins (top panels) and hR6 serum against the *C. jejuni* glycan (bottom panels). Arrows denote aglycosylated (g0) and singly glycosylated (g1) forms of the acceptor proteins. Molecular weight (MW) markers are indicated at left. Results are representative of at least three biological replicates

Given the open nature of cell-free biosynthesis, we postulated that it should be possible to functionally interchange and prototype alternative biochemical reaction components. One straightforward way that this can be accomplished is by combining separately prepared extracts, each of which is selectively enriched with a given enzyme, such that the resulting reaction mixture comprises a functional biological pathway^20,22^. As proof of this concept, separately prepared *Cj*LLO and *Cj*PglB extracts were mixed and subsequently primed with DNA encoding the scFv13-R4^DQNAT^ acceptor. The resulting mixture promoted efficient glycosylation of scFv13-R4^DQNAT^ as observed in Western blots probed with anti-His antibody and hR6 serum (Fig. 4a). In addition to scFv13-R4^DQNAT^, we also expressed a different model acceptor protein that was created by grafting a 21-amino acid sequence from the *C. jejuni* glycoprotein AcrA^34^, which was further modified with an optimized DQNAT glycosylation site, into a flexible loop of superfolder GFP (sfGFP^217-DQNAT^). The mixed lysate reaction scheme was able to glycosylate the sfGFP^217-DQNAT^ acceptor protein with 100% conversion (Fig. 4a). It is noteworthy that the high conversion observed for both acceptor proteins was achieved in mixed lysates without the need to supplement the reactions with purified OST or organic solvent-extracted *Cj*LLOs.

Next, we sought to demonstrate that the mixed lysate approach could be used to rapidly prototype the activity of four additional bacterial OSTs. Crude extracts were separately prepared from CLM24 source strains heterologously overexpressing one of the following bacterial OSTs: *Campylobacter coli* PglB (*Cc*PglB), *Desulfovibrio desulfuricans* PglB (*Dd*PglB), *Desulfovibrio gigas* PglB (*Dg*PglB), or *Desulfovibrio vulgaris* PglB (*Dv*PglB). The resulting extracts were selectively enriched with full-length OST proteins at levels that were comparable to *Cj*PglB (Supplementary Fig. 3a). Each OST extract was mixed with the *Cj*LLO-enriched extract and then supplemented with plasmid DNA encoding sfGFP^217-DQNAT^ or a modified version of this target protein where the residue in the −2 position of the acceptor sequon was mutated to alanine. Upon completion of CFGpS reactions, the expression and glycosylation status of sfGFP^217-DQNAT^ and sfGFP^217-AQNAT^ was followed by western blot analysis, which revealed information about the sequon preferences for these homologous enzymes. For example, the mixed lysate containing *Cc*PglB was observed to efficiently glycosylate sfGFP^217-DQNAT^ but not sfGFP^217-AQNAT^ (Fig. 4b). This activity profile for *Cc*PglB was identical to that observed for *Cj*PglB, which was not surprising based on its high sequence similarity (~81%) to *Cj*PglB. In contrast, lysate mixtures containing OSTs from *Desulfovibrio* sp., which have low sequence identity (~15-20%) to *Cj*PglB, showed more relaxed sequon preferences (Fig. 4b). Specifically, *Dg*PglB-enriched extract mixtures modified both (D/A)QNAT motifs with nearly equal efficiency while mixed lysates containing *Dd*OST and *Dv*OST preferentially glycosylated the AQNAT sequon.

### One-pot extract promotes biosynthesis of diverse glycoproteins

**Fig. 5:**
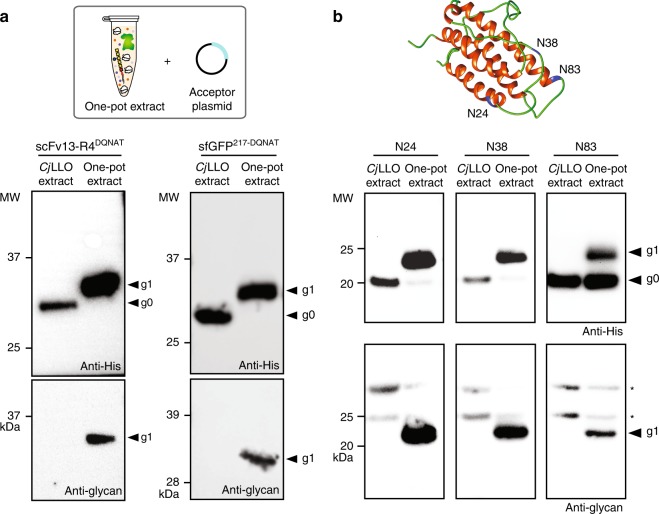
One-pot CFGpS using extracts selectively enriched with OSTs and LLOs. **a** Western blot analysis of scFv13-R4^DQNAT^ or sfGFP^217-DQNAT^ produced by crude CLM24 extract selectively enriched with (i) *Cj*PglB from heterologous overexpression from pSF-*Cj*PglB and (ii) *Cj*LLOs from heterologous overexpression from pMW07-pglΔB. Reactions were primed with plasmid pJL1-scFv13-R4^DQNAT^ or pJL1-sfGFP^217-DQNAT^. **b** Ribbon representation of human erythropoietin (PDB code 1BUY) with α-helixes and flexible loops colored in red and green, respectively. Glycosylation sites modeled by mutating the native sequons at N24 (22-AENIT-26), N38 (36-NENIT-40), or N83 (81-LVNSS-85) to DQNAT, with asparagine residues in each sequon colored in blue. Image prepared using UCSF Chimera package.^68^ Glycoengineered hEPO variants in which the native sequons at N24 (22-AENIT-26), N38 (36-NENIT-40), or N83 (81-LVNSS-85) were individually mutated to an optimal bacterial sequon, DQNAT (shown in blue). Western blot analysis of hEPO glycovariants produced by crude CLM24 extract selectively enriched with (i) *Cj*PglB from heterologous overexpression from pSF-*Cj*PglB and (ii) *Cj*LLOs from heterologous overexpression from pMW07-pglΔB. Reactions were primed with plasmid pJL1-hEPO^22-DQNAT-26^ (N24), pJL1-hEPO^36-DQNAT-40^ (N38), or pJL1-hEPO^81-DQNAT-85^ (N83) as indicated. All control reactions (lane 1 in each panel) were performed using *Cj*LLO-enriched extracts that lacked *Cj*PglB. Blots were probed with anti-hexa-histidine antibody (anti-His) to detect the acceptor proteins or hR6 serum (anti-glycan) to detect the *N-*glycan. Arrows denote aglycosylated (g0) and singly glycosylated (g1) forms of the protein targets. Asterisks denote bands corresponding to non-specific serum antibody binding. Molecular weight (MW) markers are indicated at left. Results are representative of at least three biological replicates (see Supplementary Fig. 4 for replicate data)

To create a fully integrated CFGpS platform that permits one-pot synthesis of *N-*glycoproteins without the need for supplementation of either purified OSTs or solvent-extracted LLOs (Fig. 1), we produced crude S30 extract from CLM24 cells heterologously overexpressing *Cj*PglB and the *C. jejuni* glycan biosynthesis enzymes. The resulting extract was selectively enriched with both *Cj*PglB and *Cj*LLOs donor to an extent that was indistinguishable from the separately prepared extracts (Supplementary Fig. 3a and b). Using this extract, CFGpS reactions were performed by addition of plasmid DNA encoding either scFv13-R4^DQNAT^ or sfGFP^217-DQNAT^. In both cases, 100% protein glycosylation was achieved without the need for exogenous supplementation of separately prepared glycosylation components (Fig. 5a). Independent extract preparations yielded identical results for both protein substrates, confirming the reproducibility of the CFGpS system (Supplementary Fig. 4a and b). Importantly, the in vitro synthesized scFv13-R4^DQNAT^ and sfGFP^217-DQNAT^ proteins retained biological activity that was unaffected by *N-*glycan addition (Supplementary Figs. 5 and 6). From the activity data, the yield of glycosylated scFv13-R4^DQNAT^ and sfGFP^217-DQNAT^ proteins produced by the one-pot CFGpS system was determined to be ~20 and ~10 mg L^−1^, respectively.

To determine whether human glycoproteins could be similarly produced in our one-pot system, we constructed plasmids for cell-free expression of human erythropoietin (hEPO) glycovariants in which the native sequons at residue N24 (22-AENIT-26), N38 (36-NENIT-40) or N83 (81-LVNSS-85) were individually mutated to the optimal bacterial sequon, DQNAT (Fig. 5b). CFGpS reactions were then initiated by priming the all-in-one extract with plasmid DNA encoding hEPO^22-DQNAT-26^, hEPO^36-DQNAT-40^, or hEPO^81-DQNAT-85^. Western blot analysis revealed clearly detectable glycosylation of each hEPO glycovariant with 100% glycosylated product for the N24 and N38 sites and ~30–40% for the N83 site (Fig. 5b). As with the model glycoproteins scFv13-R4^DQNAT^ and sfGFP^217-DQNAT^ above, all three glycosylated hEPO variants retained biological activity that was indistinguishable from the activity measured for the corresponding aglycosylated counterparts, with yields in the ~10 mg L^−1^ range (Supplementary Fig. 7). Collectively, these findings establish that one-pot CFGpS extracts are capable of co-activating protein synthesis and *N*-glycosylation in a manner that yields efficiently glycosylated proteins including those of human origin.

## Discussion

In this work, we successfully created a technology for one-pot biosynthesis of *N-*linked glycoproteins in the absence of living cells. This was accomplished by uniting cell-free transcription and translation with the necessary reaction components for *N-*linked protein glycosylation through a process of crude extract enrichment. By preparing OST- and LLO-enriched crude S30 extracts from a glyco-optimized chassis strain, glycosylation-competent lysates were capable of supplying efficiently glycosylated target proteins, with conversion levels at or near 100% in most instances. The glycoprotein yields obtained for three structurally diverse proteins were in the 10–20 mg L^−1^ range, which compare favorably to some of the yields reported previously for these proteins in different CFPS kits or in-house generated extracts. For example, Jackson et al.^46^ produced 3.6 mg L^−1^ of GFP using the PURExpress system, Stech et al.^47^ produced ~12 mg L^−1^ of an anti-SMAD2 scFv using a CHO cell-derived lysate, Ahn et al.^48^ produced 55 mg L^−1^ of hEPO using an *E. coli*-derived S30 lysate, and Gurramkonda et al.^31^ produced ~120 mg L^−1^ of hEPO using a CHO cell-derived lysate supplemented with CHO microsomes.

Furthermore, this work represents the first demonstration of extract enrichment with catalytically active multipass transmembrane enzymes (and their corresponding lipid-linked substrates) without the need for domain truncation or supplementation of extra scaffold molecules^49^, and provides a blueprint for other CFPS-based applications beyond glycosylation that involve this important class of proteins. Moreover, the ability of OST- or LLO-enriched crude extracts to co-activate glycosylation partially bypassed the need for costly, labor-intensive preparation of glycosylation components and paved the way for a modular single-pot CFGpS platform in which protein synthesis and *N*-linked glycosylation were integrated.

A major advantage of the CFGpS system developed here is the level of control it affords over each of the glycosylation components (i.e., catalysts, substrates, and cofactors) in terms of important process variables such as relative concentration, timing of addition, overall reaction time, etc. Likewise, genome engineering of the chassis strain used to supply the extract, such as our recent report enhancing cell-free synthesis containing multiple, identical non-canonical amino acids^16^, makes it possible to eliminate inhibitory substances such as glycosidases that catalyze the undesired hydrolysis of glycosidic linkages. This user-level control provides an opportunity to overcome system bottlenecks that effectively limit glycosylation efficiency as we showed with both the *C. jejuni* heptasaccharide and the eukaryotic Man_3_GlcNAc_2_ glycan. Moreover, the open nature of the CFGpS system could be further exploited in the future to introduce components that may otherwise be incompatible with chassis strain expression such as unusual and/or non-natural LLOs that cannot be assembled or flipped in vivo.

An additional advantage of the CFGpS system is that it does not rely on commercial cell-free kits to support protein synthesis. For comparison, the glycoproteins yields obtained here were ~10–20 mg L^−1^ in reactions costing ~$0.01–0.03 per μL (Supplementary Table 1 and also ref. ^50^) versus previous kit-based (e.g., Promega L110; NEB^®^ E6800S) glycoprotein yields of ~100 mg L^−1^ ^34^ in reactions costing ~$1 μL^−1^ ^51^. As a result, our system can synthesize ~1 μg glycoprotein/$ reagents compared to the previously published approach that can synthesize ~0.1 μg glycoprotein/$ reagents, representing an order of magnitude improvement in relative protein synthesis yields. It is also worth noting that this cost analysis does not take into account the cost of purifying OSTs or extracting LLOs that were used to supplement the commercial kits in our previous work^34^. We anticipate this reduction in cost will encourage adoption of the CFGpS platform.

Perhaps the most important feature of the CFGpS platform is its modularity, which was evidenced by the interchangeability of: (i) OST enzymes from different bacterial species; (ii) engineered LLOs with glycan moieties derived from bacteria and eukaryotes; and (iii) diverse acceptor protein targets including naturally occurring human *N-*glycoproteins with terminal or internal acceptor sequons. Importantly, enriched extracts could be readily mixed in a manner that enabled screening of an OST panel whose activities in CFGpS were in line with previously reported activities in vivo^52^, thereby validating this lysate mixing strategy as a useful tool for rapid characterization of glycosylation enzyme function and for prototyping glycosylation reactions. In light of this modularity, we envision that lysate enrichment could be further expanded beyond the glycosylation components/substrates tested here. For example, extracts could be heterologously enriched with alternative membrane-bound or soluble OSTs that catalyze *N-*linked or *O-*linked glycosyl transfer reactions. Such biocatalyst swapping is expected to be relatively straightforward in light of the growing number of prokaryotic and eukaryotic OST enzymes that have been recombinantly expressed in functional conformations and used to promote in vitro glycosylation reactions^18,49,52–56^. Likewise, as newly engineered glycan biosynthesis pathways emerge^57^, these could be readily integrated into the CFGpS platform through heterologous expression of GTs in the chassis strain. The ability to modularly reconfigure and quickly interrogate glycosylation systems in vitro should make the CFGpS technology a useful new addition to the glycoengineering toolkit for increasing our understanding of glycosylation and, in the future, advancing applications of on demand biomolecular manufacturing^58–61^.

## Methods

### Bacterial strains and plasmids

The following *E. coli* strains were used in this study: DH5α, BL21(DE3) (Novagen), CLM24, and Origami2(DE3) *gmd*::kan Δ*waaL*. DH5α was used for plasmid cloning and purification. BL21(DE3) was used for expression and purification of the scFv13-R4^DQNAT^ acceptor protein that was used in all in vitro glycosylation reactions. CLM24 is a glyco-optimized derivative of W3110 that carries a deletion in the gene encoding the WaaL ligase, thus facilitating the accumulation of preassembled glycans on Und-PP^36^. CLM24 was used for purification of the *Cj*OST enzyme, organic solvent-based extraction of all LLO sbearing bacterial glycans, and the source strain for preparing extracts with and without selectively enriched glycosylation components. Origami2(DE3) *gmd*::kan Δ*waaL* was used for producing Man_3_GlcNAc_2_-bearing LLOs and was generated by sequential mutation with P1*vir* phage transduction using the respective strains from the Keio collection^62^ as donors, which were obtained from the Coli Genetic Stock Center (CGSC). In brief, donor lysate was generated from strain JW3597-1 (Δ*rfaL734*::kan) and the resulting phage was used to infect Origami2(DE3) target cells. After plating transformants on LB plates containing kanamycin (Kan), successful transductants were selected and their Kan resistance cassettes were removed by transforming with temperature-sensitive plasmid pCP20^63^. The resulting strain, Origami2(DE3) Δ*waaL*, was then used for subsequent deletion of the *gmd* gene according to an identical strategy but using donor strain JW2038-1 (Δ*gmd751*::kan).

All plasmids used in the study are listed in Supplementary Table 2. Plasmids constructed in this study were made using standard cloning protocols and confirmed by DNA sequencing. These included the following. Plasmid pJL1-scFv13-R4^DQNAT^ was generated by first PCR amplifying the gene encoding scFv13-R4^DQNAT^ from pET28a-scFv13-R4(N34L, N77L)^DQNAT^, where the N34L and N77L mutations were introduced to eliminate putative internal glycosylation sites in scFv13-R4^52^. The resulting PCR product was then ligated between *Nco*I and *Sal*I restriction sites in plasmid pJL1, a pET-based vector used for CFPS^49^. Plasmid pJL1-sfGFP^217-DQNAT^ was generated by ligating a commercially-synthesized DNA fragment encoding sfGFP^217-DQNAT^ (Integrated DNA Technologies) into pJL1. This version of sfGFP contains an additional GT insertion after K214, which extends this flexible loop before the final beta sheet^64^. Into this flexible loop, immediately after T216, we grafted a 21-amino acid sequence containing the *C. jejuni* AcrA N123 glycosylation site^34^, but with an optimal DQNAT sequon in place of the native AcrA sequon. Similar procedures were used to generate plasmids pJL1-sfGFP^217-AQNAT^, pJL1-hEPO^22-DQNAT-26^, pJL1-hEPO^36-DQNAT-40^, and pJL1-hEPO^81-DQNAT-85^. In the case of pJL1-hEPO^22-DQNAT-26^, the gene for mature human EPO was designed such that the native sequon at N24 was changed from 22-AENIT-26 to an optimal bacterial sequon, DQNAT. Identical cloning strategies were carried out to separately introduce optimal DQNAT motifs in place of the native hEPO sequons 36-NENIT-40 and 81-LVNSS-85. Recombinant expression of the *E. coli* O9 primer-adaptor glycan (Man_3_GlcNAc) on Und-PP was achieved by cloning the genes encoding the WbdB and WbdC mannosyltransferase enzymes derived from *E. coli* ATCC31616 for assembling the glycan, and RfbK and RfbM, also derived from *E. coli* ATCC31616 for increasing the pool of available GDP-mannose, in *E. coli* MG1655. Plasmid pConYCGmCB was constructed by isothermal Gibson assembly and encodes an artificial operon comprised of: (i) the yeast glycosyltransferases Alg13, Alg14, Alg1, and Alg2 for Man_3_GlcNAc_2_ glycan biosynthesis^12^ and (ii) the *E. coli* enzymes phosphomannomutase (ManB) and mannose-1-phosphate guanylyltransferase (ManC), which together increase availability of GDP-mannose substrates for the Alg1 and Alg2 enzymes.

### Protein expression and purification

Purification of *Cj*PglB was performed according to a previously described protocol^34^. Briefly, a single colony of *E. coli* CLM24 carrying plasmid pSN18^65^ was grown overnight at 37 °C in 50 mL of Luria-Bertani (LB; 10 g L^−1^ tryptone, 5 g L^−1^ yeast extract, 5 g L^−1^ NaCl, pH 7.2) supplemented with ampicillin (Amp) and 0.2% (w/v%) d-glucose. Overnight cells were subcultured into 1 L of fresh terrific broth (TB; 12 g L^−1^ tryptone, 24 g L^−1^ yeast extract, 0.4% (v/v%) glycerol, 10% (v/v%) 0.17 M KH_2_PO_4_/0.72 M K_2_HPO_4_ phosphate buffer), supplemented with Amp and grown until the absorbance at 600 nm (Abs_600_) reached a value of ~0.7. The incubation temperature was adjusted to 16 °C, after which protein expression was induced by the addition of l-arabinose to a final concentration of 0.02% (w/v%). Protein expression was allowed to proceed for 20 h at 16 °C. Cells were harvested by centrifugation and then disrupted using a homogenizer (Avestin C5 EmulsiFlex). The lysate was centrifuged to remove cell debris and the supernatant was ultracentrifuged (100,000×*g*) for 2 h at 4 °C. The resulting pellet containing the membrane fraction was fully resuspended with a Potter-Elvehjem tissue homogenizer in buffer containing 50 mM HEPES, 250 mM NaCl, 10% (v/v%) glycerol, and 1% (w/v%) *n*-dodecyl-β-d-maltoside (DDM) at pH 7.5. The suspension was incubated at room temperature for 1 h to facilitate detergent solubilization of *Cj*PglB from native *E. coli* lipids, which were removed by subsequent ultracentrifugation (100,000×*g*) for 1 h at 4 °C. The supernatant containing DDM-solubilized *Cj*PglB was purified using Ni-NTA resin (Thermo) according to manufacturer’s specification with the exception that all buffers were supplemented with 1% (w/v%) DDM. The elution fraction from Ni-NTA purification was then subjected to size exclusion chromatography (SEC) using an ÄKTA Explorer FPLC system (GE Healthcare) with Superdex 200 10/300 GL column. Purified protein was stored at a final concentration of 1–2 mg mL^−1^ in OST storage buffer (50 mM HEPES, 100 mM NaCl, 5% (v/v%) glycerol, 0.01% (w/v%) DDM, pH 7.5) at 4 °C. Glycerol concentration in the sample was adjusted to 20% (v/v%) for long-term storage at −80 °C.

Purification of acceptor protein scFv13-R4^DQNAT^ was carried out as described previously^52^. Briefly, *E. coli* strain BL21(DE3) carrying plasmid pET28a-scFv13-R4(N34L, N77L)^DQNAT^ was grown in 1 L of TB supplied with kanamycin. The culture was incubated at 37 °C until Abs_600_ reached ~0.7, at which point protein expression was induced by addition of isopropyl β-d-1-thiogalactopyranoside (IPTG) to a final concentration of 0.1 mM. Protein expression was allowed to proceed for 20 h at 25 °C. Cells were harvested and disrupted identically as described above. The scFv13-R4^DQNAT^ protein was purified using Ni-NTA resin followed by SEC according to manufacturer’s protocols. Protein was stored at a final concentration of 1–2 mg mL^−1^ in storage buffer (50 mM HEPES, 250 mM NaCl, 1 mM EDTA, pH 7.5) at 4 °C.

### Extraction of LLOs

The protocol for organic solvent extraction of LLOs from *E. coli* membranes was adapted from a previously described protocol^34,66^. In most cases, a single colony of strain CLM24 carrying a plasmid for target glycan biosynthesis (Supplementary Table 2) was grown overnight in LB media. The notable exceptions were LLOs bearing the *W. succinogenes N-*glycan (*Ws*LLOs), which were produced using DH5α cells carrying the pEpiFOS-5*pgl5* fosmid (kindly provided by Dr. Markus Aebi), and LLO sbearing Man_3_GlcNAc_2_, which were produced using Origami2(DE3) *gmd*::kan Δ*waaL* cells carrying plasmid pConYCGmCB. Overnight cells were subcultured into 1 L of TB supplemented with an appropriate antibiotic and grown until the Abs_600_ reached ~0.7. The incubation temperature was adjusted to 30 °C for biosynthesis of all glycans except for Man_3_GlcNAc_2_, which was adjusted to 16 °C. For plasmid pMW07-pglΔB, protein expression was induced with l-arabinose at a final concentration of 0.2% (w/v%) while for fosmid pEpiFOS-5*pgl5* induction was with isopropyl β-d-1-thiogalactopyranoside (IPTG) at a final concentration of 1 mM. All other plasmids involved constitutive promoters and thus did not require chemical inducers. After 16 h, cells were harvested by centrifugation and cell pellets were lyophilized to complete dryness at −70 °C. For extraction of *Cj*LLOs, native and engineered *Cl*LLOs, *E. coli* O9 primer-adaptor LLOs, and *Ws*LLOs, the lyophilisates were suspended in 10:20:3 volumetric ratio of CHCl_3_:CH_3_OH:H_2_O solution and incubated at room temperature for 15 min to facilitate extraction of LLOs. For extraction of LLO sbearing Man_3_GlcNAc_2_ glycan, lyophilisate was successively suspended in 10:20 (v/v%) CHCl_3_:CH_3_OH solution, water, and 10:20:3 CHCl_3_:CH_3_OH:H_2_O solution with 15 min of incubation at room temperature between each step. In each case, the final suspension was centrifuged (4000×*g*) for 15 min, after which the organic layer (bottom layer) was collected and dried with a vacuum concentrator followed by lyophilization. Lyophilisates containing active LLOs were resuspended in cell-free glycosylation buffer (10 mM HEPES, pH 7.5, 10 mM MnCl_2_, and 0.1% (w/v%) DDM) and stored at 4 °C.

### Preparation of crude S30 extracts

CLM24 source strains were grown in 2×YTPG (10 g L^−1^ yeast extract, 16 g L^−1^ tryptone, 5 g L^−1^ NaCl, 7 g L^−1^ K_2_HPO_4_, 3 g L^−1^ KH_2_PO_4_, 18 g L^−1^ glucose, pH 7.2) until the Abs_600_ reached ~3. To generate OST-enriched extract, CLM24 carrying plasmid pSF-*Cj*PglB, pSF-*Cc*PglB, pSF-*Dd*PglB, pSF-*Dg*PglB, or pSF-*Dv*PglB^52^ was used as the source strain. To generate LLO-enriched extract, CLM24 carrying plasmid pMW07-pglΔB was used as the source strain. To generate one-pot extract containing both OST and LLOs, CLM24 carrying pMW07-pglΔB and pSF-*Cj*OST was used as the source strain. As needed, the expression of glycosylation components was induced with l-arabinose at final concentration of 0.02% (w/v%). After induction, protein expression was allowed to proceed at 30 °C to a density of OD_600_ ~3, at which point cells were harvested by centrifugation (5000×*g*) at 4 °C for 15 min. All subsequent steps were carried out at 4 °C unless otherwise stated. Pelleted cells were washed three times in S30 buffer (10 mM tris acetate, 14 mM magnesium acetate, 60 mM potassium acetate, pH 8.2). After the last wash, cells were pelleted at 7000×*g* for 10 min and flash-frozen on liquid nitrogen. To make lysate, cells were thawed and resuspended to homogeneity in 1 mL of S30 buffer per 1 g of wet cell mass. Cells were disrupted using an Avestin EmulsiFlex-B15 high-pressure homogenizer at 20,000–25,000 psi with a single passage. The lysate was then centrifuged twice at 30,000×*g* for 30 min to remove cell debris. Supernatant was transferred to a new vessel and incubated with 250 rpm shaking at 37 °C for 60 min to degrade endogenous mRNA transcripts and disrupt existing polysome complexes in the lysate. Following centrifugation (15,000×*g*) for 15 min at 4 °C, supernatant was collected, aliquoted, flash-frozen in liquid nitrogen, and stored at −80 °C. S30 extract was active for about three freeze-thaw cycles and contained ~40 g L^−1^ total protein as measured by Bradford assay.

### Cell-free glycoprotein synthesis

For in vitro glycosylation of purified acceptor protein, reactions were carried out in a 50 μL volume containing 3 μg of scFv13-R4^DQNAT^, 2 μg of purified *Cj*PglB, and 5 μg extracted LLOs (in the case of Man_3_GlcNAc_2_ LLOs, 20 μg was used) in in vitro glycosylation buffer (10 mM HEPES, pH 7.5, 10 mM MnCl_2_, and 0.1% (w/v%) DDM). The reaction mixture was incubated at 30 ° C for 16 h. For crude extract-based expression of glycoproteins, a two-phase scheme was implemented. In the first phase, protein synthesis was carried out with a modified PANOx-SP system^67^. Specifically, 1.5 mL microcentrifuge tubes were charged with 15-µL reactions containing 200 ng plasmid DNA, 30% (v/v%) S30 extract and the following: 12 mM magnesium glutamate, 10 mM ammonium glutamate, 130 mM potassium glutamate, 1.2 mM adenosine triphosphate (ATP), 0.85 mM guanosine triphosphate (GTP), 0.85 mM uridine triphosphate (UTP), 0.85 mM cytidine triphosphate (CTP), 0.034 mg mL^−1^ folinic acid, 0.171 mg mL^−1^ *E. coli* tRNA (Roche), 2 mM each of 20 amino acids, 30 mM phosphoenolpyruvate (PEP, Roche), 0.4 mM nicotinamide adenine dinucleotide (NAD), 0.27 mM coenzyme-A (CoA), 4 mM oxalic acid, 1 mM putrescine, 1.5 mM spermidine, and 57 mM HEPES. For scFv13-R4^DQNAT^, hEPO^22-DQNAT-26^, hEPO^36-DQNAT-40^, and hEPO^81-DQNAT85^, this phase was carried out at 30 °C for 4 h under oxidizing conditions while for sfGFP^217-DQNAT^ and sfGFP^217-AQNAT^ this phase was carried out at 30 °C for 5 min under reducing conditions. For oxidizing conditions, extract was pre-conditioned with 750 μM iodoacetamide in the dark at room temperature for 30 min and the reaction mix was supplied with 200 mM glutathione at a 3:1 ratio between oxidized and reduced forms. The active sfGFP yields from cell-free reactions were quantified by measuring fluorescence in-lysate and converting into concentration using a standard curve as previously described^39^. In the second phase, protein glycosylation was initiated by the addition of MnCl_2_ and DDM at a final concentration of 10 mM and 0.1% (w/v%), respectively, and allowed to proceed at 30 °C for 16 h. As needed, reactions were supplemented with 2 μg of purified *Cj*PglB (i.e., for CFGpS with LLO-enriched extracts) or 5 μg solvent-extracted *Cj*LLOs (i.e., for CFGpS with OST-enriched extracts). All reactions were stopped by adding Laemmli sample buffer containing 5% βME, after which samples were boiled at 100 °C for 15 min and analyzed by SDS-PAGE and western blotting.

### Western blot analysis

Samples containing 0.5 μg of acceptor protein were loaded into SDS-PAGE gels. Following electrophoretic separation, proteins were transferred from gels onto Immobilon-P polyvinylidene difluoride (PVDF) membranes (0.45 μm) according to manufacturer’s protocol. Membranes were washed twice with TBS buffer (80 g L^−1^ NaCl, 20 g L^−1^ KCl, and 30 g L^−1^ Tris-base) followed by incubation for 1 h in blocking solution (50 g L^−1^ non-fat milk in TBST (TBS supplied with 0.05% (v/v%) Tween-20)). After blocking, membranes were washed 4 times with TBST with 10 min incubation between each wash. A first membrane was probed with 6xHis-polyclonal antibody (Abcam, ab137839, 1:7500) that specifically recognizes hexahistidine epitope tags while a second replicate membrane was probed with one of the following: hR6 (1:10,000) serum from rabbit that recognizes the native *C. jejuni* and *C. lari* glycan as well as engineered *C. lari* glycan or ConA-HRP (Sigma, L6397, 1:2500) that recognizes Man_3_GlcNac and Man_3_GlcNAc_2_. Probing of membranes was performed for at least 1 h with shaking at room temperature, after which membranes were washed with TBST in the same manner as described above. For development, membranes were incubated briefly at room temperature with Western ECL substrate (BioRad) and imaged using a ChemiDocTM XRS+System. OST enzymes enriched in extracts were detected by an identical SDS-PAGE procedure followed by Western blot analysis with a polyclonal antibody specific to the FLAG epitope tag (Abcam, ab49763, 1:7500). The glycan component of LLOs enriched in extracts was detected by directly spotting 10 μL of extracts onto nitrocellulose membranes followed by detection with hR6 serum. Uncropped immunoblot images are shown in Supplementary Fig. 8.

### MS analysis

Approximately 2 μg of scFv13-R4^DQNAT^ protein in solution was denatured with 6 M urea, reduced with 10 mM DTT, incubated at 34 °C for 1 h, then alkylated with 58 mM iodoacetamide for 45 min in the dark at room temperature and quenched by final 36 mM DTT. The solution was then diluted with 50 mM ammonium bicarbonate (pH 8.0) to a final buffer concentration of 1 M urea prior to trypsin digestion. Sample was digested with 0.2 μg of trypsin for 18 h at 37 °C. The digestion was stopped by addition of TFA to a final pH 2.2–2.5. The samples were then desalted with SOLA HRP SPE Cartridge (ThermoFisher Scientific). The cartridges were conditioned with 1 × 0.5 mL 90% methanol, 0.1% trifluoroacetic acid (TFA) and equilibrated with 2 × 0.5 mL 0.1% (v/v%) TFA. The samples were diluted 1:1 with 0.2% (v/v%) TFA and run slowly through the cartridges. After washing with 2 × 0.5 mL of equilibration solution, peptides were eluted by 1 × 0.5 mL of 50% (v/v%) acetonitrile (ACN), 0.1% (v/v%) TFA and dried in a speed vacuum centrifuge.

The nanoLC–MS/MS analysis was carried out using UltiMate3000 RSLCnano (Dionex) coupled to an Orbitrap Fusion (ThermoFisher Scientific) mass spectrometer equipped with a nanospray Flex Ion Source. Each sample was reconstituted in 22 µL of 0.5% (w/v%) FA and 10 μL was loaded onto an Acclaim PepMap 100 C18 trap column (5 µm, 100 µm × 20 mm, 100 Å, ThermoFisher Scientific) with nanoViper Fittings at 20 μL min^−1^ of 0.5% FA for on-line desalting. After 2 min, the valve switched to allow peptides to be separated on an Acclaim PepMap C18 nano column (3 µm, 75 µm × 25 cm, ThermoFisher Scientific), in a 90 min gradient of 5 to 23% to 35% B at 300 nL min^−1^ (3 to 73 to 93 min, respectively), followed by a 9-min ramping to 90% B, a 9-min hold at 90% B and quick switch to 5% B in 1 min. The column was re-equilibrated with 5% B for 20 min prior to the next run. The Orbitrap Fusion was operating in positive ion mode with nanospray voltage set at 1.7 kV and source temperature at 275 °C. External calibration for FT, IT and quadrupole mass analyzers was performed prior to the analysis. The Orbitrap full MS survey scan (*m*/*z* 400–1800) was followed by Top 3 s data-dependent Higher Collision dissociation product ion triggered ETD (HCD-pd-ETD) MS/MS scans for precursor peptides with 2–7 charges above a threshold ion count of 50,000 with normalized collision energy of 32%. MS survey scans were acquired at a resolving power of 120,000 (FWHM at *m*/*z* 200), with Automatic Gin Control (AGC) = 2e5 and maximum injection time (Max IT) = 50 ms, and HCD MS/MS scans at a resolution of 30,000 with AGC = 5e4, Max IT = 60 ms and with Q isolation window (*m*/*z*) at 3 for the mass range *m*/*z* 105–2000. Dynamic exclusion parameters were set at 1 within 60 s exclusion duration with ±10 ppm exclusion mass width. Product Ion trigger list consisted of peaks at 204.0867 Da (HexNAc oxonium ion), 138.0545 Da (HexNAc fragment), and 366.1396 Da (HexHexNAc oxonium ions). If one of the HCD product ions in the list was detected, two charge-dependent ETD MS/MS scans (EThcD) with HCD supplemental activation (SA) on the same precursor ion were triggered and collected in a linear ion trap. For doubly charged precursors, the ETD reaction time as set 150 ms and the SA energy was set at 30%, while the same parameters at 125 ms and 20%, respectively, were used for higher charged precursors. For both ion triggered scans, fluoranthene ETD reagent target was set at 2e5, AGC target at 1e4, Max IT at 105 ms and isolation window at 3. All data were acquired using Xcalibur 3.0 operation software and Orbitrap Fusion Tune Application v. 2.1 (ThermoFisher Scientific).

All MS and MS/MS raw spectra from each sample were searched using Byonics v. 2.8.2 (Protein Metrics) using the *E coli* protein database with added scFv13-R4^DQNAT^ protein target sequence. The peptide search parameters were as follows: two missed cleavage for full trypsin digestion with fixed carbamidomethyl modification of cysteine, variable modifications of methionine oxidation, and deamidation on asparagine/glutamine residues. The peptide mass tolerance was 10 ppm and fragment mass tolerance values for HCD and EThcD spectra were 0.05 and 0.6 Da, respectively. Both the maximum number of common and rare modifications were set at two. The glycan search was performed against a list of 309 mammalian *N*-linked glycans in Byonic software. Identified peptides were filtered for maximum 2% FDR. The software exported the results of the search to a spreadsheet.

### GFP fluorescence activity

The activity of cell-free-derived sfGFP was determined using an in-lysate fluorescence analysis as described previously^39^. Briefly, 2 μL of cell-free synthesized glycosylated sfGFP reaction was diluted into 48 μL of nanopure water. The solution was then placed in a Costar 96-well black assay plate (Corning). Excitation and emission wavelengths for sfGFP fluorescence were 485 and 528 nm, respectively.

### Enzyme-linked immunosorbent analysis (ELISA)

Costar 96-well ELISA plates (Corning) were coated overnight at 4 °C with 50 μl of 1 mg mL^−1^ *E. coli* β-gal (Sigma-Aldrich) in 0.05 M sodium carbonate buffer (pH 9.6). After blocking with 5% (w/v%) bovine serum albumin (BSA) in PBS for 3 h at room temperature, the plates were washed four times with PBST buffer (PBS, 0.05% (v/v%) Tween-20, 0.3% (w/v%) BSA) and incubated with serially diluted purified scFv13-R4 samples or soluble fractions of CFGpS lysates for 1 h at room temperature. Samples were quantified by the Bradford assay and an equivalent amount of total protein was applied to the plate. After washing four times with the same buffer, anti-6×-His-HRP conjugated rabbit polyclonal antibody (Abcam) in 3% PBST was added to each well for 1 h. Plates were washed and developed using standard protocols.

### In vitro cell proliferation assay

Human erythroleukemia TF-1 cells (Sigma) that require granulocyte–macrophage colony-stimulating factor (GM-CSF), interleukin 3 (IL-3), or hEPO for growth and survival were used. Cells were maintained in RPMI-1640 media supplemented with 10% FBS, 50 U mL^−1^ penicillin, 50 mg mL^−1^ streptomycin, 2 mM glutamine, and 2 ng mL^−1^ GM-CSF at 37 °C in a humidified atmosphere containing 5% CO_2_. After 16 h incubation in RPMI-1640 media without GM-CSF, cells were counted, harvested, and resuspended in fresh media. 5 × 10^3^ TF-1 cells per well were seeded in a 96-well assay plate, and EPO standards or samples were added to final desired concentrations to each well. Cells were incubated with for 6 h in humid incubator before adding alamarBlue^®^. After 12 h, fluorescence signal was measured at 560 nm/590 nm excitation/emission wavelength.

### Data availability

All data generated during the study are included in this article and its supplementary information files, and are available from the authors upon reasonable request.

## Electronic supplementary material

Supplementary Information
