# Author Correction: Single-pot glycoprotein biosynthesis using a cell-free transcription-translation system enriched with glycosylation machinery

**DOI:** 10.1038/s41467-018-05620-8

**Published:** 2018-08-20

**Authors:** Thapakorn Jaroentomeechai, Jessica C. Stark, Aravind Natarajan, Cameron J. Glasscock, Laura E. Yates, Karen J. Hsu, Milan Mrksich, Michael C. Jewett, Matthew P. DeLisa

**Affiliations:** 1 Robert Frederick Smith School of Chemical and Biomolecular Engineering, Cornell University, Ithaca, NY 14853 USA; 2 Department of Chemical and Biological Engineering, Northwestern University, Evanston, IL 60208 USA; 3 Chemistry of Life Processes Institute, 2170 Campus Drive, Evanston, IL 60208- 3120 USA; 4 Center for Synthetic Biology, Northwestern University, 2145 Sheridan Road, Evanston, IL 60208- 3120 USA; 5 Department of Microbiology, Cornell University, Ithaca, NY 14853 USA; 6 Department of Mechanical Engineering, Northwestern University, 2145 Sheridan Rd Technological Institute B224, Evanston, IL 60208-3120 USA; 7 Department of Chemistry, Northwestern University, Evanston, IL 60208 USA; 8 Department of Cell and Molecular Biology, Northwestern University, Chicago, IL 60611 USA; 9 Department of Biomedical Engineering, Northwestern University, Evanston, IL 60208 USA

Correction to: *Nature Communications*; 10.1038/s41467-018-05110-x; published online 12-July-2018

The original version of this Article contained an error in Figure 2, wherein the bottom right western blot panel in Figure 2a was blank. This has now been corrected in both the PDF and HTML versions of the Article.

