# Supplementary material for: Single-pot glycoprotein biosynthesis using a cell-free transcription-translation system enriched with glycosylation machinery
Source: Nat Commun. 2018 Jul 12;9:2686. doi: 10.1038/s41467-018-05110-x (PMC6043479; doi:10.1038/s41467-018-05110-x)
Supplement: Supplementary file 1 — Supplementary Information [file 41467_2018_5110_MOESM1_ESM.pdf]

Supplementary Information

Single-pot glycoprotein biosynthesis using a cell-free transcription-translation system enriched with glycosylation machinery

Thapakorn Jaroentomeechai, Jessica C. Stark, Aravind Natarajan, Cameron J. Glasscock, Laura E. Yates, Karen J. Hsu, Milan Mrksich, Michael C. Jewett, and Matthew P. DeLisa

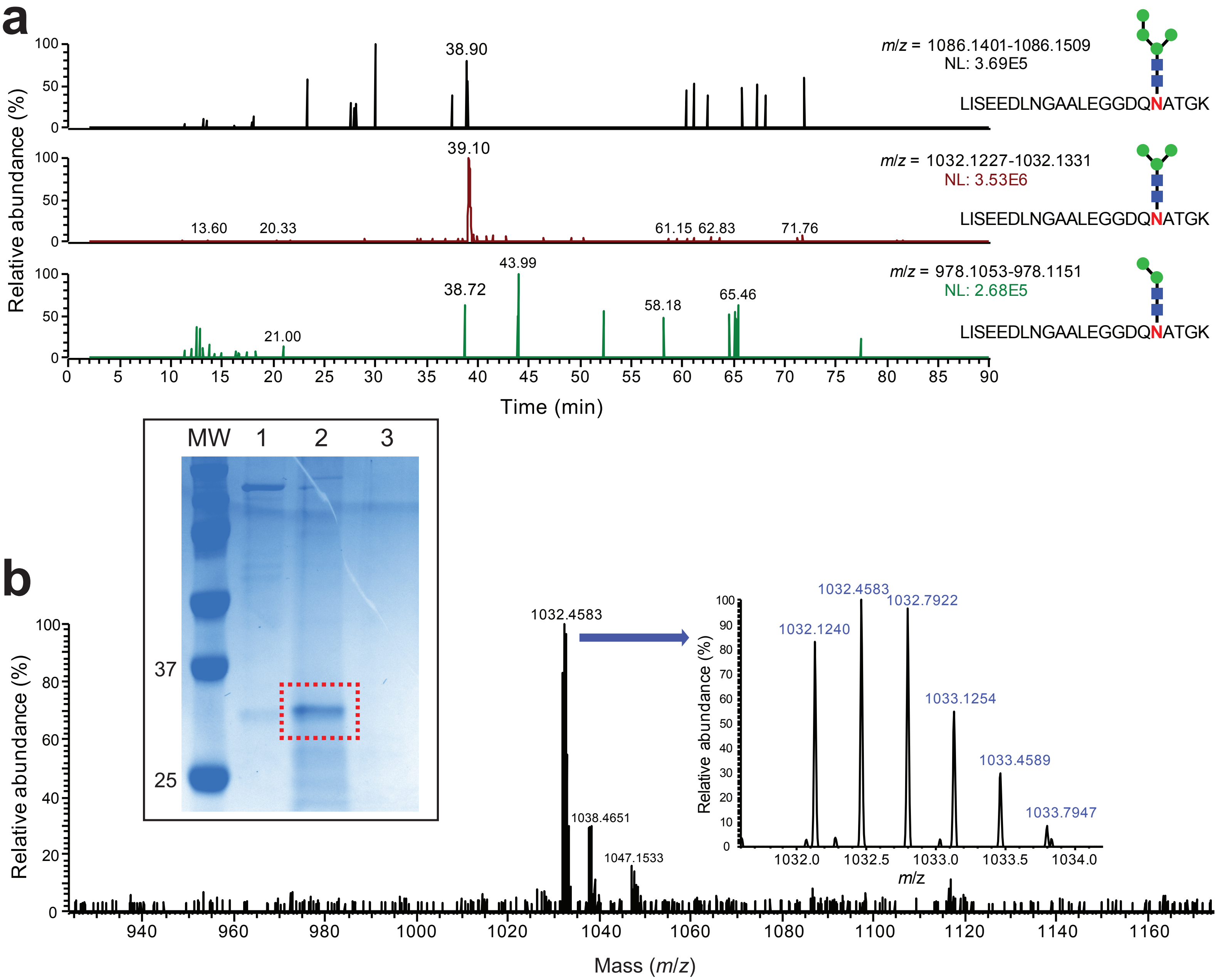

Supplementary Figure 1. MS analysis of scFv13-R4^{DQNAT} glycosylated with $Man_3GlcNAc_2$. Ni-NTA-purified scFv13-R4^{DQNAT} was subjected to *in vitro* glycosylation in the presence of purified *Cj*PglB and organic solvent-extracted $Man_3GlcNAc_2$ LLOs, and then directly loaded into an SDS-PAGE gel. Following staining of gel with Coomassie Brilliant Blue G-250 (inset), the glycosylated band (lane 2, indicated by red box) was excised and submitted for MS analysis. Controls included *in vitro* glycosylation reaction performed with solvent-extracted empty LLOs (lane 1) and complete *in vitro* glycosylation reaction mixture lacking purified scFv13-R4^{DQNAT} acceptor protein (lane 3). Molecular weight (MW) ladder loaded on the left. **(a)** Three extracted ion chromatograms (XIC) corresponding to mass ranges for three possible glycopeptide products having masses consistent with the expected $Man_3GlcNAc_2$ (middle), as well as $Man_4GlcNAc_2$ (top) and $Man_2GlcNAc_2$ (bottom) attached to N273 site of scFv13-R4^{DQNAT} (mass tolerance at 5 ppm). The individually normalized level (NL) for each glycoform indicates that only a $Hex_3HexNAc_2$ glycoform, which eluted at 39.10 min with NL of 3.53E6, was decently detected in the sample (middle). A trace amount of a $Hex_4HexNAc_2$ glycoform form eluted at 38.9 min with NL of 2.96E5 (top), but no $Hex_2HexNAc_2$ glycoform was detected. **(b)** MS spectrum of the detected glycopeptide containing an *N*-linked pentasaccharide consistent with $Man_3GlcNAc_2$ at m/z = 1032.4583. The MS inset shows an expanded view of the glycopeptide ion with triple charge.

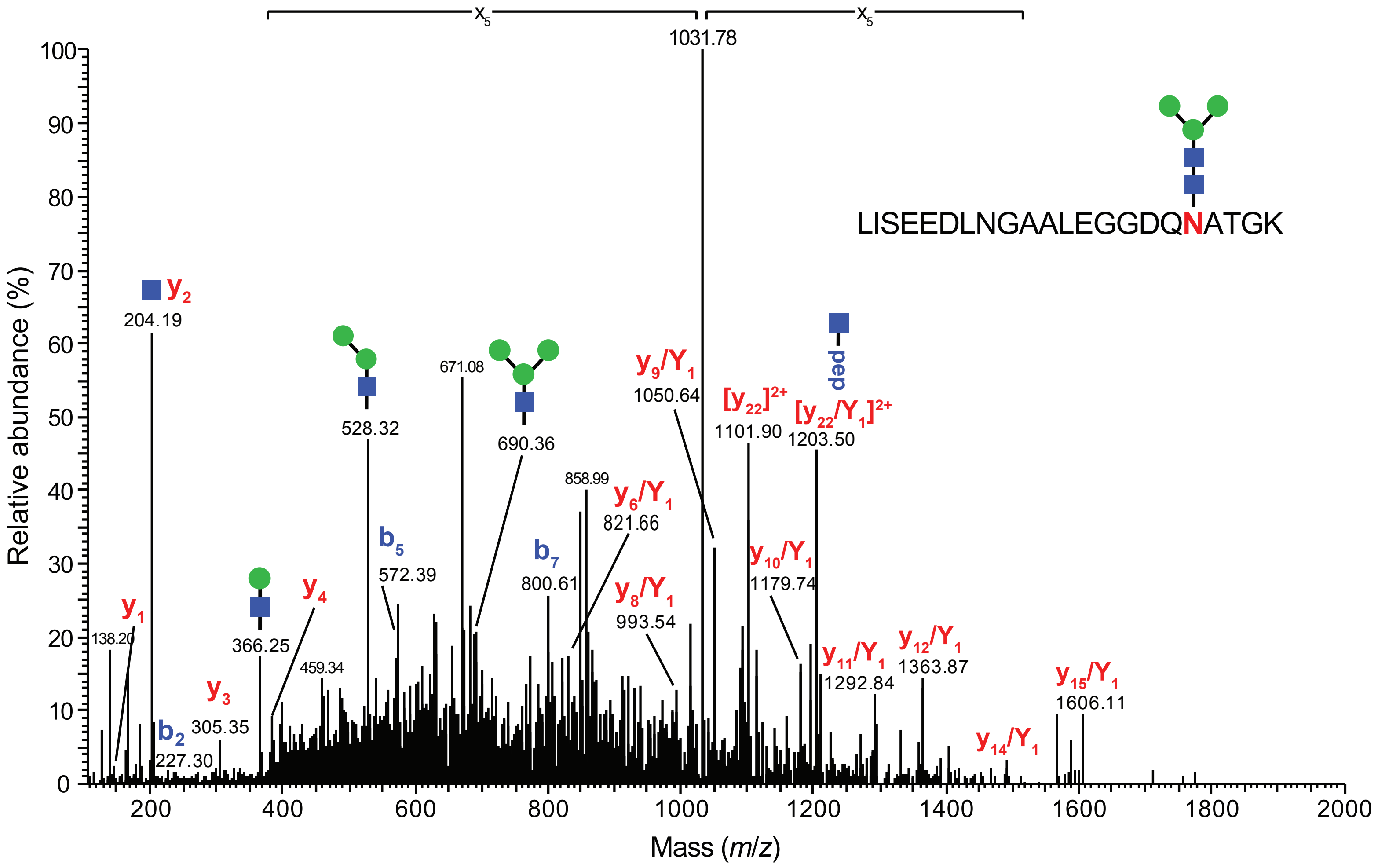

Supplementary Figure 2. Tandem mass spectrometry of scFv13-R4^{DQNAT} glycosylated with Man$_3$GlcNAc$_2$. MS/MS spectrum of the triply-charged precursor (*m/z* 1032.12), identifying the glycopeptide with core pentasaccharide (Hex$_3$HexNAc$_2$) attached to residue N273 (shown in red) in scFv13-R4^{DQNAT}. A series of y-ions covering from y1 to y4 and a second series of y-ions with the added mass of 203.08 Da at N273 site were found covering from y6/Y1 to y15/Y1, leading to the confident identification of tryptic peptide 256-LISEEDLNGAALEGGDQNATGK-277 and providing direct evidence for HexNAc as the innermost monosaccharide (Y1) attached to the N273 site. This result is also consistent with the previous observation that a relatively tight bond exists for the Y1-peptide compared to the fragile internal glycan bonds.

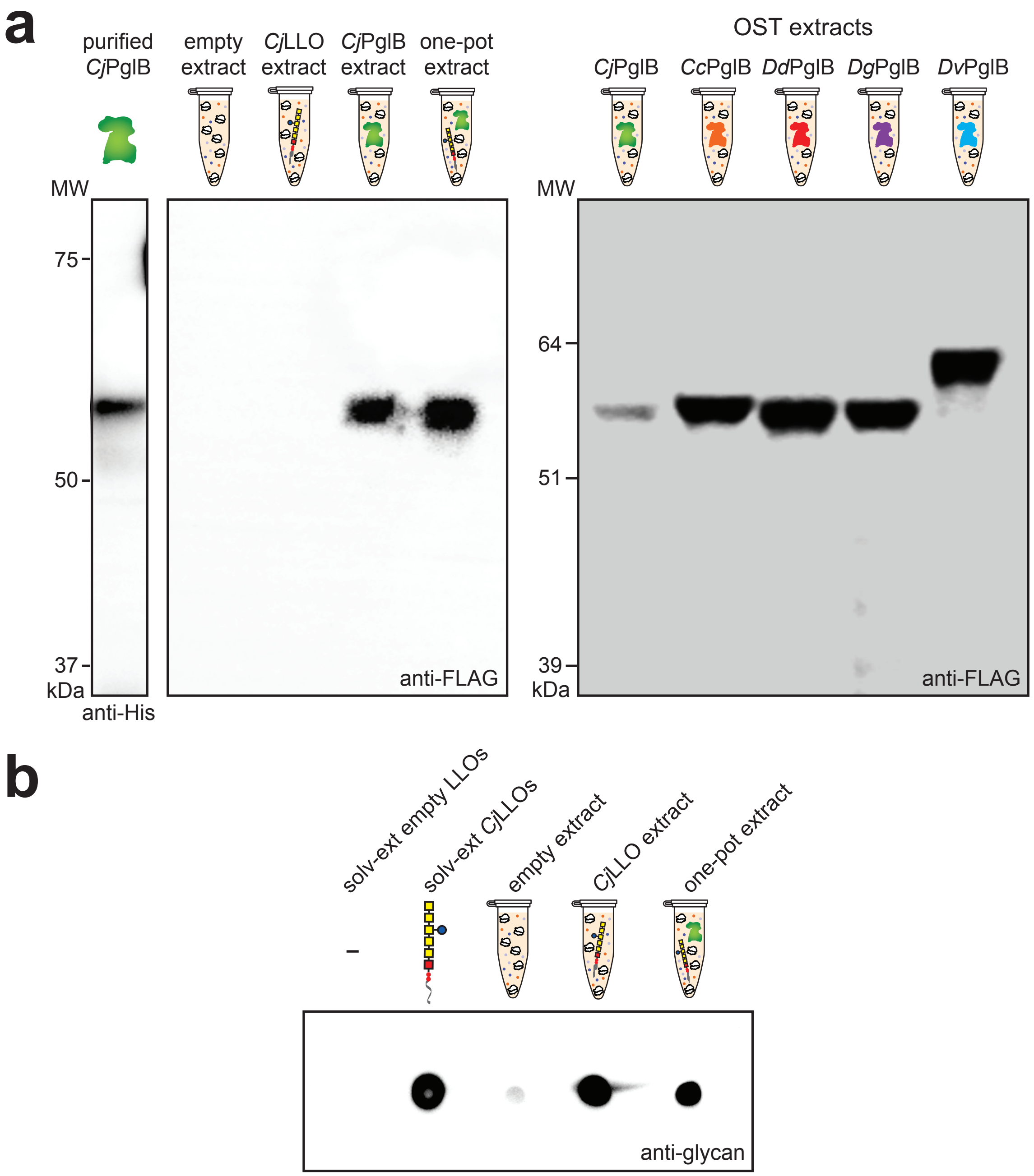

Supplementary Figure 3. Crude cell extracts are enriched with glycosylation machinery. (a) Western blot analysis of *Cj*PglB in the following samples: (left-hand panel) 1 µg of purified *Cj*PglB; (center panel) crude cell extracts derived from CLM24 cells with no plasmid (empty extract), CLM24 cells carrying pMW07-pglΔB (*Cj*LLO extract), CLM24 cells carrying pSF-*Cj*PglB (*Cj*PglB extract) or CLM24 cells carrying pMW07-pglΔB and pSF-*Cj*PglB (one-pot extract); and (right-hand panel) crude cell extracts derived from CLM24 cells carrying pSF-based plasmids encoding different PglB homologs as indicated. Blots were probed with anti-His antibody and anti-FLAG antibody as indicated. Molecular weight (MW) markers are indicated at left. Results are representative of at least three biological replicates. **(b)** Dot blot analysis of LLOs in the following samples: organic solvent extract from membrane fractions of CLM24 cells with no plasmid (solv-ext empty LLOs) or from CLM24 cells carrying plasmid pMW07-pglΔB (solv-ext *Cj*LLOs); crude cell extracts derived from CLM24 cells with no plasmid (empty extract), CLM24 cells carrying pMW07-pglΔB (*Cj*LLO extract) or CLM24 cells carrying pMW07-pglΔB and pSF-*Cj*PglB (one-pot extract). 10 µl of extracted LLOs or crude cell extract was spotted onto nitrocellulose membrane and probed with hR6 serum (anti-glycan).

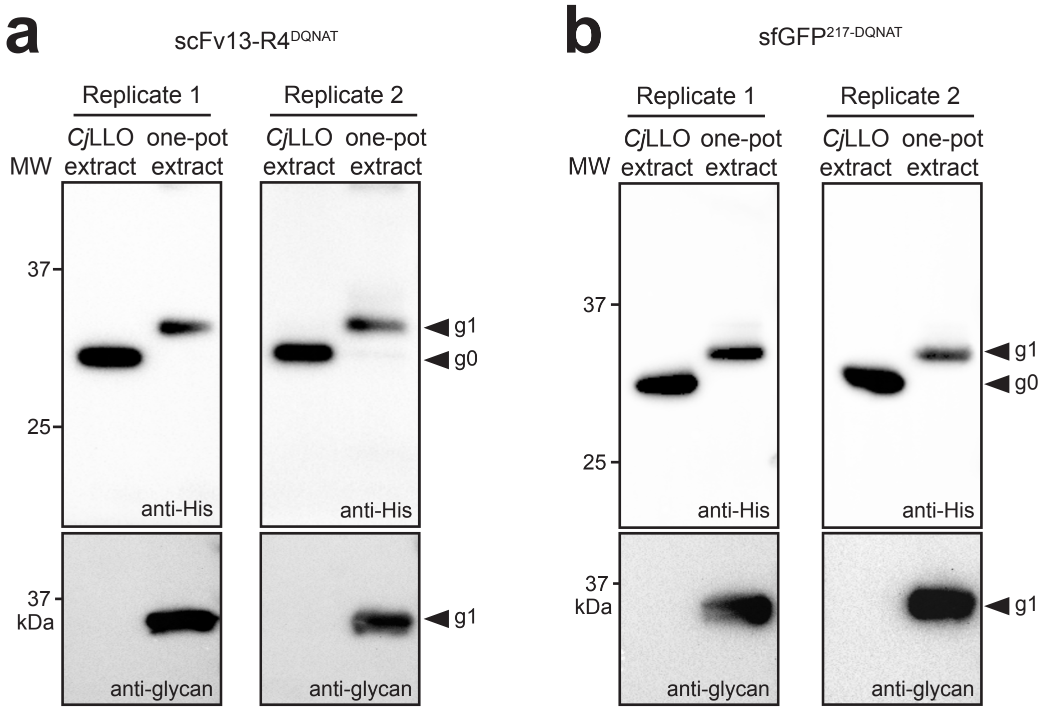

Supplementary Figure 4. Independent biological replicates for one-pot CFGpS reactions. Western blot analysis replicated twice for both the **(a)** scFv13-R4^{DQNAT} and **(b)** sfGFP$^{217\text{-}DQNAT}$ acceptor proteins produced using crude CLM24 extract selectively enriched with (i) *Cj*PglB from heterologous overexpression from pSF-*Cj*PglB and (ii) *Cj*LLOs from heterologous overexpression from pMW07-pglΔB. Each replicate experiment involved charging freshly prepared cell-free extracts with freshly purified pJL1-scFv13-R4^{DQNAT} or pJL1-sfGFP$^{217\text{-}DQNAT}$ plasmid DNA. Control reactions (lane 1 in each panel) were performed using *Cj*LLO-enriched extracts that lacked *Cj*PglB. Blots were probed with anti-hexa-histidine antibody (anti-His) to detect acceptor proteins or hR6 serum (anti-glycan) to detect the *N*-glycan. Arrows denote aglycosylated (g0) and singly glycosylated (g1) forms of the protein targets. Molecular weight (MW) markers are indicated at left.

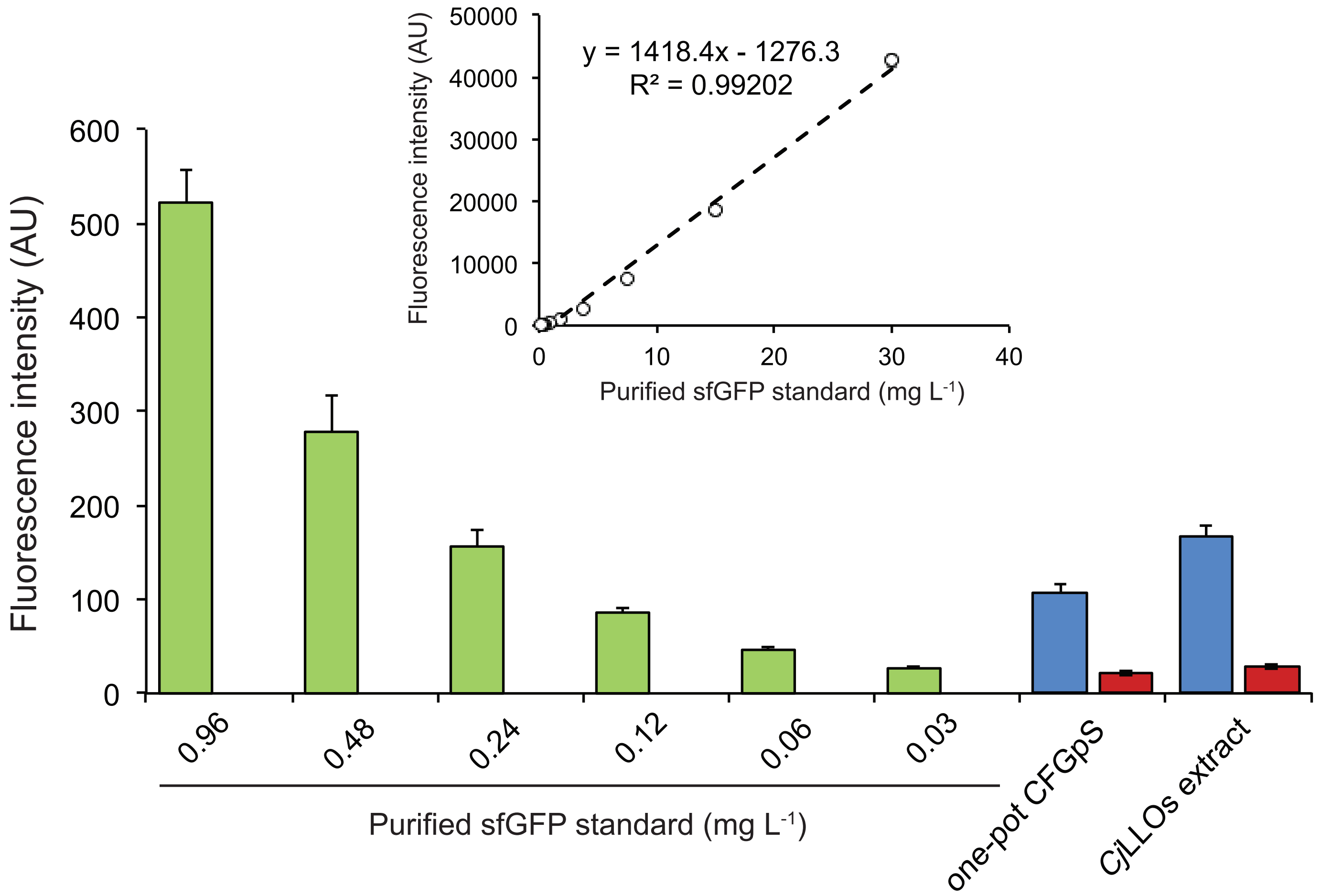

Supplementary Figure 5. CFGpS expression of active sfGFP. In-lysate fluorescence activity for glycosylated (one-pot CFGpS) and aglycosylated (*Cj*LLOs extract) sfGFP$^{217\text{-}DQNAT}$ produced in cell-free reactions charged with plasmid pJL1-sfGFP$^{217\text{-}DQNAT}$ (blue) or with no plasmid DNA (red). Following 2-h reactions, cell-free reactions containing glycosylated and aglycosylated sfGFP$^{217\text{-}DQNAT}$ were diluted 10 times with water and then subjected to fluorescence measurement. Excitation and emission wavelengths for sfGFP were 485 and 528 nm, respectively. Calibration curve was prepared by measuring fluorescence intensity of aglycosylated sfGFP$^{217\text{-}DQNAT}$ expressed and purified from *E. coli* cells and mixed with empty extract. Linear regression analysis (inset) was used to calculate the concentration of glycosylated sfGFP$^{217\text{-}DQNAT}$ in the samples, which was determined to be ~10 mg L^{-1}. Data are the average of three biological replicates and error bars represent the standard deviation of the mean.

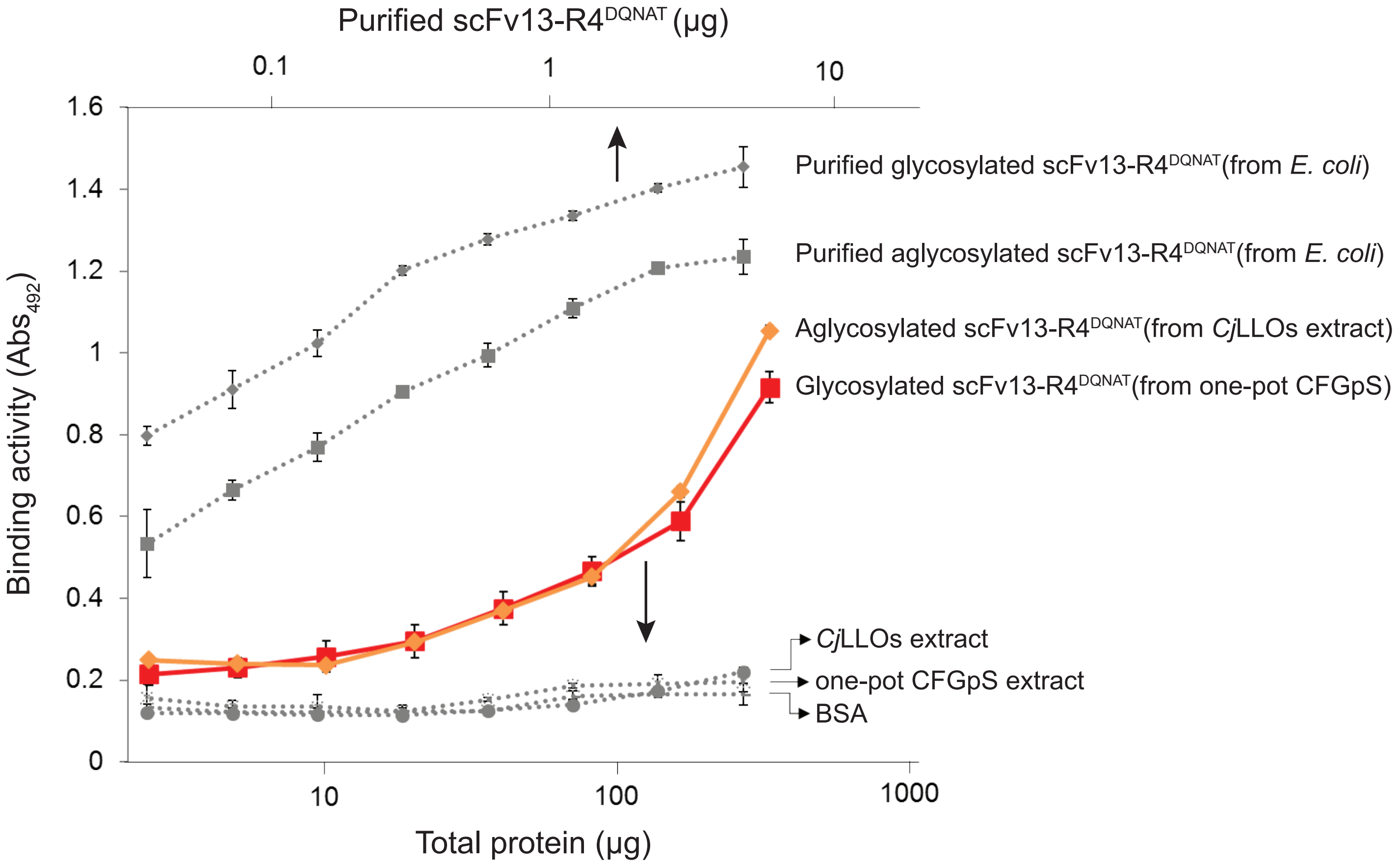

Supplementary Figure 6. CFGpS expression of active scFv antibody fragment. Antigen-binding activity for β-gal-specific scFv13-R4^{DQNAT} measured by ELISA with *E. coli* β-gal as immobilized antigen. The scFv13-R4^{DQNAT} acceptor was produced as a glycosylated protein in one-pot CFGpS (red) or an aglycosylated protein in control extracts containing *Cj*LLOs but not *Cj*PglB (orange). Extracts were primed with plasmid pJL1- scFv13-R4^{DQNAT}. Positive controls included the same scFv13-R4^{DQNAT} protein produced *in vivo* by recombinant expression in *E. coli* in the presence (glycosylated) or absence (aglycosylated) of glycosylation machinery. Negative controls included extracts without plasmid and BSA. Comparing to signals from purified protein, the concentration of glycosylated scFv13-R4^{DQNAT} was determined to be ~20 mg L^{-1}. Data are the average of three biological replicates and error bars represent the standard deviation of the mean.

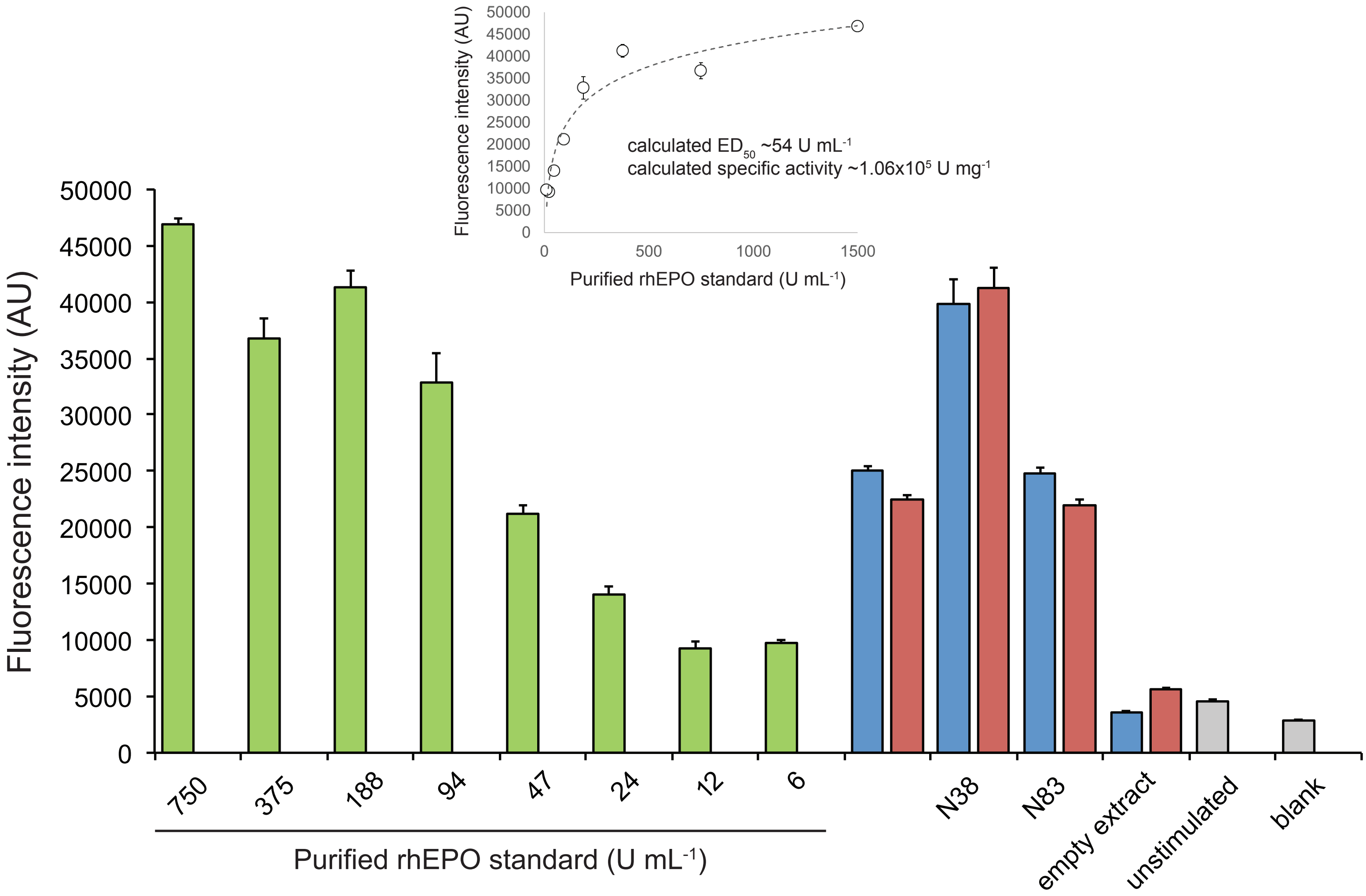

Supplementary Figure 7. CFGpS-derived hEPO glycovariants stimulate cell proliferation. Stimulation of human erythroleukemia TF-1 cell proliferation following incubation with purified rhEPO standard or hEPO variants produced in cell-free reactions. For CFGpS-derived hEPO glycovariants, TF-1 cells were treated with either glycosylated hEPO variants produced in one-pot CFGpS (blue) or aglycosylated hEPO variants produced in control extracts containing *Cj*LLOs but not *Cj*PglB (red). To produce the hEPO variants, extracts were primed with plasmid pJL1-hEPO$^{22\text{-DQNAT-}26}$ (N24), pJL1-hEPO$^{36\text{-DQNAT-}40}$ (N38), or pJL1-hEPO$^{81\text{-DQNAT-}85}$ (N83). For positive control rhEPO samples, cells were treated with serial dilutions of commercial rhEPO that was purified from CHO cells and thus glycosylated (green). TF-1 cells incubated with empty extracts or PBS (unstimulated) served as negative controls while RPMI media without cells was used as the blank. Regression analysis (inset) was performed to determine the concentration of hEPO variants in the samples, which was found to be at ~10 mg L^{-1}. Data are the average of three biological replicates and error bars represent the standard deviation of the mean.

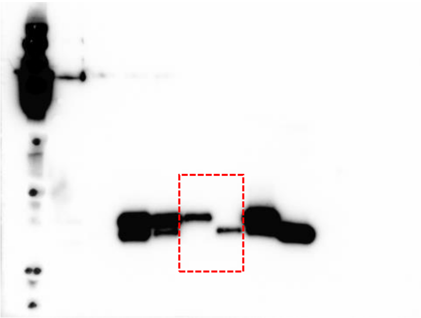

Figure 2a (left panel, anti-His blot, flipped)

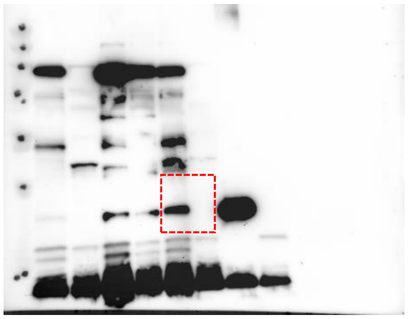

Figure 2a (left panel, anti-glycan blot, flipped)

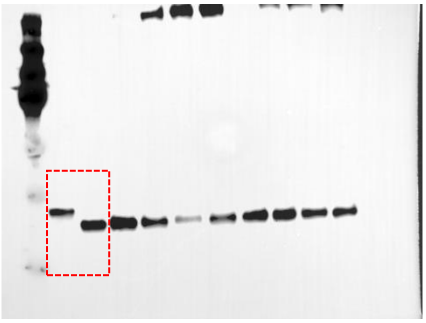

Figure 2a (right panel, anti-His blot, flipped)

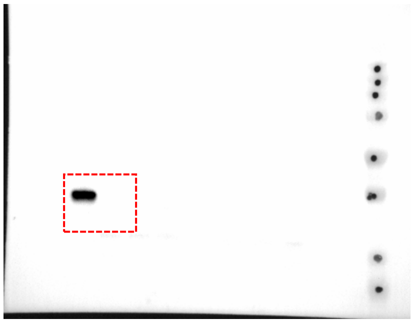

Figure 2a (right panel, anti-glycan blot, flipped)

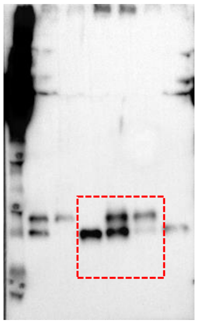

Figure 2b (left panel, anti-His blot)

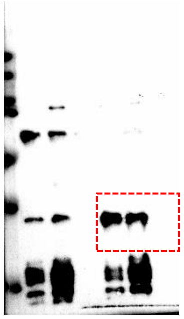

Figure 2b (left panel, anti-glycan blot)

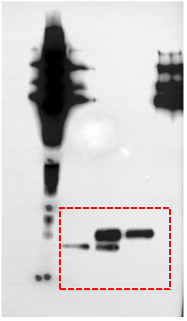

Figure 2b
(right panel, anti-His blot)

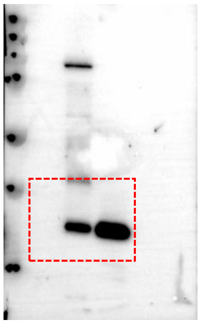

Figure 2b
(right panel, anti-glycan blot)

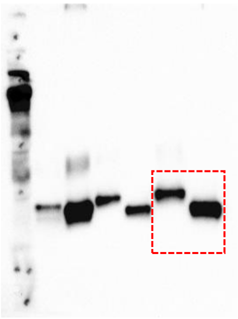

Figure 3a
(anti-His blot, flipped)

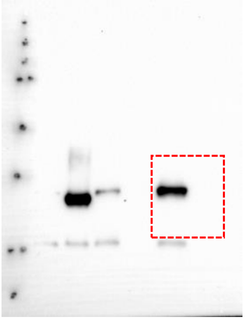

Figure 3a
(anti-glycan blot, flipped)

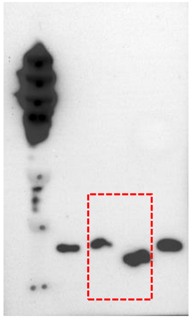

Figure 3b
(anti-His blot, flipped)

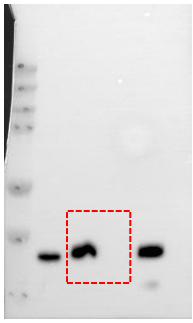

Figure 3b
(anti-glycan blot, flipped)

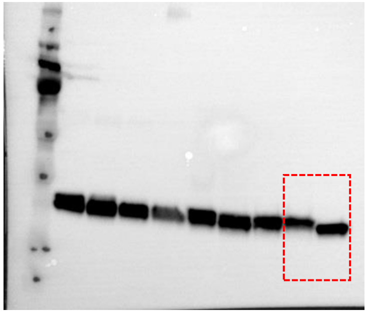

Figure 3c
(anti-His blot, flipped)

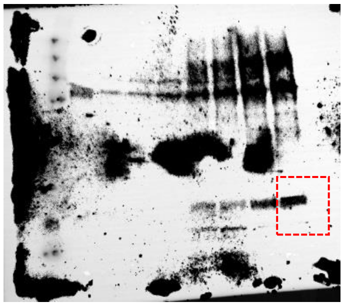

Figure 3c
(ConA blot, flipped)

Supplementary Figure 8. Full, uncropped images of immunoblots presented in the main text. Cropped areas are indicated by red boxes. Some blots were cropped and then flipped to make data presentation consistent throughout the manuscript; these cases are marked as “flipped”.

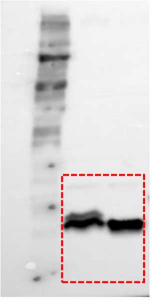

Figure 3d
(anti-His blot, flipped)

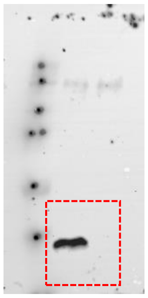

Figure 3d
(ConA blot, flipped)

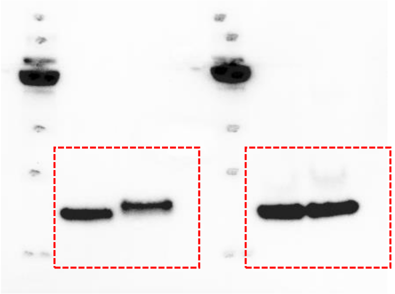

Figure 3e
(anti-His blot)

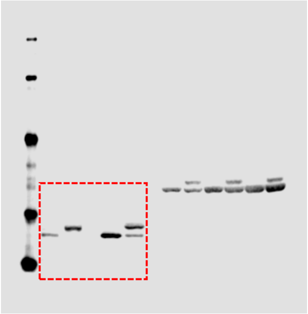

Figure 4a
(anti-His blot)

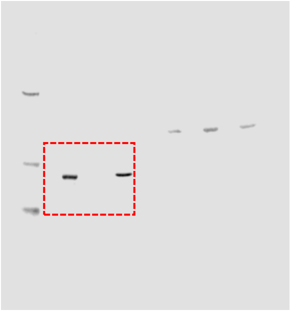

Figure 4a
(anti-glycan blot)

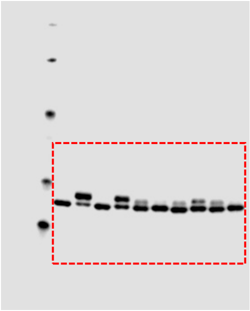

Figure 4b
(anti-His blot)

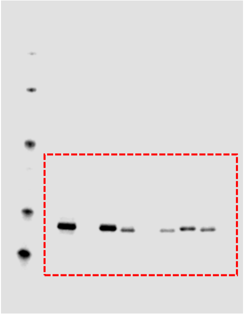

Figure 4b
(anti-glycan blot)

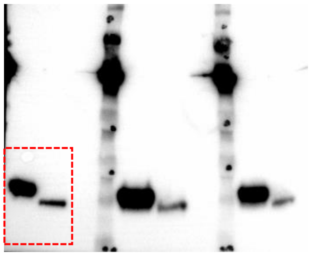

Figure 5a
(anti-His blot for scFv13-R4, flipped)

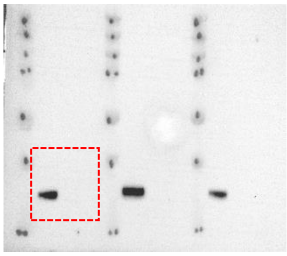

Figure 5a
(anti-glycan blot for scFv13-R4, flipped)

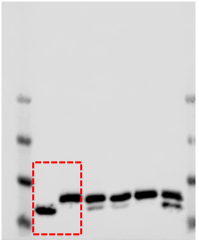

Figure 5a
(anti-His blot for sfGFP)

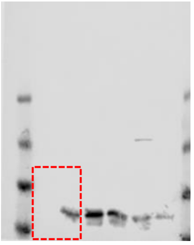

Figure 5a
(anti-glycan blot for sfGFP)

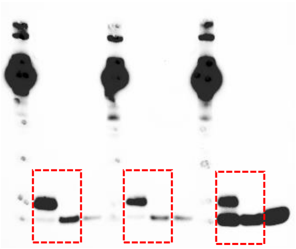

Figure 5b
(anti-His blot, flipped)

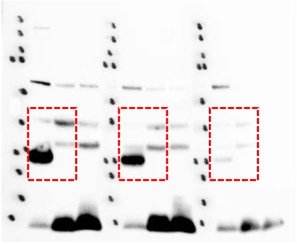

Figure 5b
(anti-glycan blot, flipped)

Supplementary Figure 8 (continued). Full, uncropped images of immunoblots presented in the main text. Cropped areas are indicated by red boxes. Some blots were cropped and then flipped to make data presentation consistent throughout the manuscript; these cases are marked as “flipped”.

Supplementary Table 1. Cost analysis of CFGpS reactions. The total cost to assemble CFGpS reactions is ~$0.01 per μL. In the table, amino acid cost accounts for 2 mM each of the 20 canonical amino acids purchased individually from Sigma. Extract cost is based on a single employee making 50 mL lysate from a 10 L fermentation, assuming 30 extract batches per year and a 5-year equipment lifetime. Component source is included in the table if it is available to purchase directly from a supplier. Homemade or user-supplied components cannot be purchased directly and must be prepared by the end user according to procedures described in the Methods section.

Component	Cost ($/μL rxn)	Supplier	Product No
$Mg(Glu)_2$	negligible	Sigma	49605
NH_4Glu	negligible	MP	02180595
KGlu	negligible	Sigma	G1501
ATP	negligible	Sigma	A2383
GTP	0.000265	Sigma	G8877
UTP	0.000230	Sigma	U6625
CTP	0.000200	Sigma	C1506
Folinic acid	0.0000206	Sigma	47612
tRNA	0.000215	Roche	10109541001
Amino acids	negligible	homemade	
PEP	0.00179	Roche	10108294001
NAD	negligible	Sigma	N8535-15VL
CoA	0.000336	Sigma	C3144
Oxalic acid	negligible	Sigma	P0963
Putrescine	negligible	Sigma	P5780
Spermidine	negligible	Sigma	S2626
HEPES	negligible	Sigma	H3375
$MnCl_2$	negligible	Sigma	63535
DDM	0.000358	Anatrace	D310S
Plasmid	negligible	user-supplied	
Extract	0.00737	homemade	
**Total**	**0.0108**	**$/μL rxn**	

Supplementary Table 2. Plasmids used in this study.

Plasmids	Description	Reference
pSN18	modified pBAD expression plasmid encoding *C. jejuni pglB* with a C-terminal decahistidine affinity tag.	[1]
pET28a-scFv13-R4 (N34L, N77L)^{DQNAT}	pET28a(+) plasmid encoding scFv13-R4 modified with a C-terminal DQNAT glycosylation tag and two mutations (N34, N77L) to eliminate putative internal glycosylation sites.	[2]
pMW07-pglΔB	pMW07 plasmid encoding *C. jejuni* protein glycosylation locus (*pgl*) with complete in-frame deletion of *Cj*PglB	[3]
pACYC*pgl2*	pACYC plasmid encoding modified *C.lari* hexasaccharide glycan biosynthesis gene cluster lacking bacillosamine biosynthesis genes	[4]
pACYC*pgl4*	pACYC plasmid encoding native *C.lari* hexasaccharide hexasaccharide glycan biosynthesis gene cluster	[5]
pEpiFOS-5*pgl5*	pEpiFOS-5 encoding the *Wolinella succinogenes* hexasaccharide glycan biosynthesis gene cluster cloned in the Eco72 site	Lab stock
pConYCG-mCB	pMW07 plasmid encoding $Man_3GlcNAc_2$ glycan biosynthesis genes and *manCB* genes for GDP-mannose biosynthesis	Lab stock
pJL1-scFv13-R4^{DQNAT}	pJL1 plasmid encoding scFv13-R4 (N34L, N77L)^{DQNAT}	This study
pJL1-sfGFP$^{217\text{-}DQNAT}$	pJL1 plasmid encoding superfolder GFP modified after residue T216 with 21 amino acid insertion containing the *C. jejuni* AcrA N123 glycosylation site but with an optimal DQNAT sequon	This study
pJL1-sfGFP$^{217\text{-}AQNAT}$	same as pJL1-sfGFP^{DQNAT} but with AQNAT sequon	This study
pJL1-hEPO$^{22\text{-}DQNAT\text{-}26}$	pJL1 plasmid encoding human erythropoietin with native glycosylation motif surrounding N22 mutated to DQNAT	This study
pJL1-hEPO$^{36\text{-}DQNAT\text{-}40}$	pJL1 plasmid encoding human erythropoietin with native glycosylation motif surrounding N38 mutated to DQNAT	This study
pJL1-hEPO$^{81\text{-}DQNAT\text{-}85}$	pJL1 plasmid encoding human erythropoietin with native glycosylation motif surrounding N83 mutated to DQNAT	This study
pSF-*Cj*PglB	pSN18 derivative encoding *C. jejuni* PglB with C-terminal FLAG epitope tag	[3]
pSF-*Cc*PglB	pSN18-derivative encoding *C. coli* PglB with C-terminal FLAG epitope tag	[3]
pSF-*Dd*PglB	pSN18-derivative encoding *D. desulfuricans* PglB with C-terminal FLAG epitope tag	[3]
pSF-*Dg*PglB	pSN18-derivative encoding *D. gigas* PglB with C-terminal FLAG epitope tag	[3]
pSF-*Dv*PglB	pSN18-derivative encoding *D. vulgaris* PglB with C-terminal FLAG epitope tag	[3]

Supplementary References

1. Kowarik, M. et al. *N*-linked glycosylation of folded proteins by the bacterial oligosaccharyltransferase. *Science* **314**, 1148-50 (2006).
2. Ollis, A.A. et al. Substitute sweeteners: diverse bacterial oligosaccharyltransferases with unique *N*-glycosylation site preferences. *Sci Rep* **5**, 15237 (2015).
3. Ollis, A.A., Zhang, S., Fisher, A.C. & DeLisa, M.P. Engineered oligosaccharyltransferases with greatly relaxed acceptor-site specificity. *Nat Chem Biol* **10**, 816-22 (2014).
4. Schwarz, F. et al. A combined method for producing homogeneous glycoproteins with eukaryotic *N*-glycosylation. *Nat Chem Biol* **6**, 264-6 (2010).
5. Schwarz, F. et al. Relaxed acceptor site specificity of bacterial oligosaccharyltransferase in vivo. *Glycobiology* **21**, 45-54 (2011).
